# The *Candida albicans* biofilm gene circuit modulated at the chromatin level by a recent molecular histone innovation

**DOI:** 10.1371/journal.pbio.3000422

**Published:** 2019-08-09

**Authors:** Laxmi Shanker Rai, Rima Singha, Hiram Sanchez, Tanmoy Chakraborty, Bipin Chand, Sophie Bachellier-Bassi, Shantanu Chowdhury, Christophe d’Enfert, David R. Andes, Kaustuv Sanyal

**Affiliations:** 1 Molecular Mycology Laboratory, Molecular Biology and Genetics Unit, Jawaharlal Nehru Centre for Advanced Scientific Research, Bangalore, India; 2 Unité Biologie et Pathogénicité Fongiques, Institut Pasteur, USC2019 INRA, Paris, France; 3 Department of Medicine, University of Wisconsin, Madison, Wisconsin, United States of America; 4 Genotypic Technology Private Limited, Bangalore, India; 5 GNR Center for Genome Informatics, CSIR-Institute of Genomics and Integrative Biology, New Delhi, India; 6 Proteomics and Structural Biology Unit, CSIR-Institute of Genomics and Integrative Biology, New Delhi, India; Fred Hutchinson Cancer Research Center, UNITED STATES

## Abstract

Histone H3 and its variants regulate gene expression but the latter are absent in most ascomycetous fungi. Here, we report the identification of a variant histone H3, which we have designated H3V^CTG^ because of its exclusive presence in the CTG clade of ascomycetes, including *Candida albicans*, a human pathogen. *C*. *albicans* grows both as single yeast cells and hyphal filaments in the planktonic mode of growth. It also forms a three-dimensional biofilm structure in the host as well as on human catheter materials under suitable conditions. H3V^CTG^ null (*hht1/hht1*) cells of *C*. *albicans* are viable but produce more robust biofilms than wild-type cells in both in vitro and in vivo conditions. Indeed, a comparative transcriptome analysis of planktonic and biofilm cells reveals that the biofilm circuitry is significantly altered in H3V^CTG^ null cells. H3V^CTG^ binds more efficiently to the promoters of many biofilm-related genes in the planktonic cells than during biofilm growth, whereas the binding of the core canonical histone H3 on the corresponding promoters largely remains unchanged. Furthermore, biofilm defects associated with master regulators, namely, biofilm and cell wall regulator 1 (Bcr1), transposon enhancement control 1 (Tec1), and non-dityrosine 80 (Ndt80), are significantly rescued in cells lacking H3V^CTG^. The occupancy of the transcription factor Bcr1 at its cognate promoter binding sites was found to be enhanced in the absence of H3V^CTG^ in the planktonic form of growth resulting in enhanced transcription of biofilm-specific genes. Further, we demonstrate that co-occurrence of valine and serine at the 31st and 32nd positions in H3V^CTG^, respectively, is essential for its function. Taken together, we show that even in a unicellular organism, differential gene expression patterns are modulated by the relative occupancy of the specific histone H3 type at the chromatin level.

## Abstract 2

A variant histone H3 specific to the CTG clade of ascomycete fungi modulates the expression of the majority of the biofilm genes in the human pathogen Candida albicans by binding differentially to biofilm-relevant gene promoters.

## Introduction

Histones are highly conserved proteins across eukaryotes. Variant histones, which are nonallelic isoforms of the canonical histones, may exist and differ from their canonical counterparts in the primary amino acid sequence and expression timing during cell cycle [1–3]. These histone variants show sequence divergence that ranges from a stretch of a few amino acids to a large domain [3–5]. Genes encoding canonical histones are usually organized in tandem multicopy clusters [6], whereas noncanonical histone variants are encoded by genes that are scattered throughout the genome [7]. Incorporation of histone variants at specific genomic loci is associated with cellular processes, including DNA replication, transcription, recombination and repair [8–10]. Several lines of evidence suggest that histone variants and their covalent post-translational modifications (PTMs) play a key role in developmental processes such as the initiation and maintenance of pericentric heterochromatin, X chromosome inactivation, and germ cell differentiation [4, 11]. Therefore, histone variants are involved in indexing the eukaryotic genome into many epigenomes. Multiple histone H3 variants are known to exist in animals, plants, and protists [4, 12]. Noncanonical histone H3 variants are also known to be present in the fungal phyla of Basidiomycota and Zoopagomycota [13]. However, it was long thought that only the canonical histone H3 is present in Ascomycota [14], a fungal phylum that includes budding yeast *Saccharomyces cerevisiae* and fission yeast *Schizosaccharomyces pombe* [3, 15]. Moreover, instead of histone H3 variant, a histone H4 variant (hH4v) is present in *Neurospora crassa* with unknown function [16].

Although unicellular, many yeast species are polymorphic in nature. A group of ascomycetes including *Candida albicans* in which CUG often codes for serine instead of leucine belong to the CTG clade. *C*. *albicans* is primarily a commensal in the oral cavity, digestive tract, and genital regions of a healthy individual [17] and yet is responsible for superficial or disseminated, often deadly infections. *C*. *albicans* undergoes high-frequency phenotypic transitions [18], among which a reversible switch from a single-celled oval yeast form to a filamentous hyphal form is thought to be required for its pathogenic lifestyle and is shown to be tightly linked with its virulence [19]. These morphological switching events are associated with global changes in transcriptional profiles that usually occur over a short time span in response to cues from the host niche.

*C*. *albicans*, like many other microbes, has the ability to form surface-associated, matrix-embedded communities called biofilms [20]. Biofilms can form both in vitro on abiotic surfaces and in vivo on biotic surfaces such as the oral and vaginal mucosa [21, 22]. Because biofilms are associated with a protective extracellular matrix, the cells in biofilms are more resistant to conventional antifungal drugs and host immune factors in comparison with the free-floating planktonic cells. Further, cross contamination through medical devices via biofilm is a major source of infection in hospitals [21, 23, 24]. Under most conditions, both yeast and filamentous hyphal cells of *C*. *albicans* are essential for proper biofilm formation. The hyphal cells provide a support scaffold required for the architectural stability of the biofilm structure formed by specific arrangements of yeast and hyphal cells. Thus, the ability of a cell to form hyphae is critical for proper biofilm growth and maintenance.

During biofilm development, cells attach to a surface and grow as a microcolony that develops into a complex three-dimensional structure held together by an extracellular matrix [25]. During biofilm growth, many adhesins, which carry a C-terminal sequence for covalent attachment of a glycosylphosphophatidylinositol (GPI) anchor, are up-regulated compared with their expression in planktonic cells [26, 27]. Some of these adhesins are Enhanced Adherence to Polystyrene 1 (Eap1) [28], Hyphal Wall Protein 1 (Hwp1) [29], Repressed By *TUP1* (Rbt1), and members of the Agglutinin-Like Sequence (Als) family [30, 31, 32]. In addition, a transcriptional network of 6 master regulators (Efg1, Tec1, Bcr1, Ndt80, Rob1, and Brg1) and approximately 1,000 target genes of these transcription factors regulate biofilm development in *C*. *albicans* both in vitro and in vivo [33]. This biofilm network is further extended by the involvement of the Flo8 Gal4, and Rfx2 transcriptional regulators [34].

In this work, we analyzed a large number of fungal genomes, and we discovered the existence of a novel variant histone H3 that is unique to the CTG clade including *C*. *albicans*. We present the molecular basis of morphological switching events at the chromatin level modulated by the canonical and variant histone H3 proteins in *C*. *albicans*. Our results reveal that this hitherto unknown CTG-clade-specific variant histone H3 is a major regulator of the biofilm gene circuitry in *C*. *albicans*.

## Results

### A unique histone H3 variant protein is encoded only by the CTG-clade species

**Fig 1:**
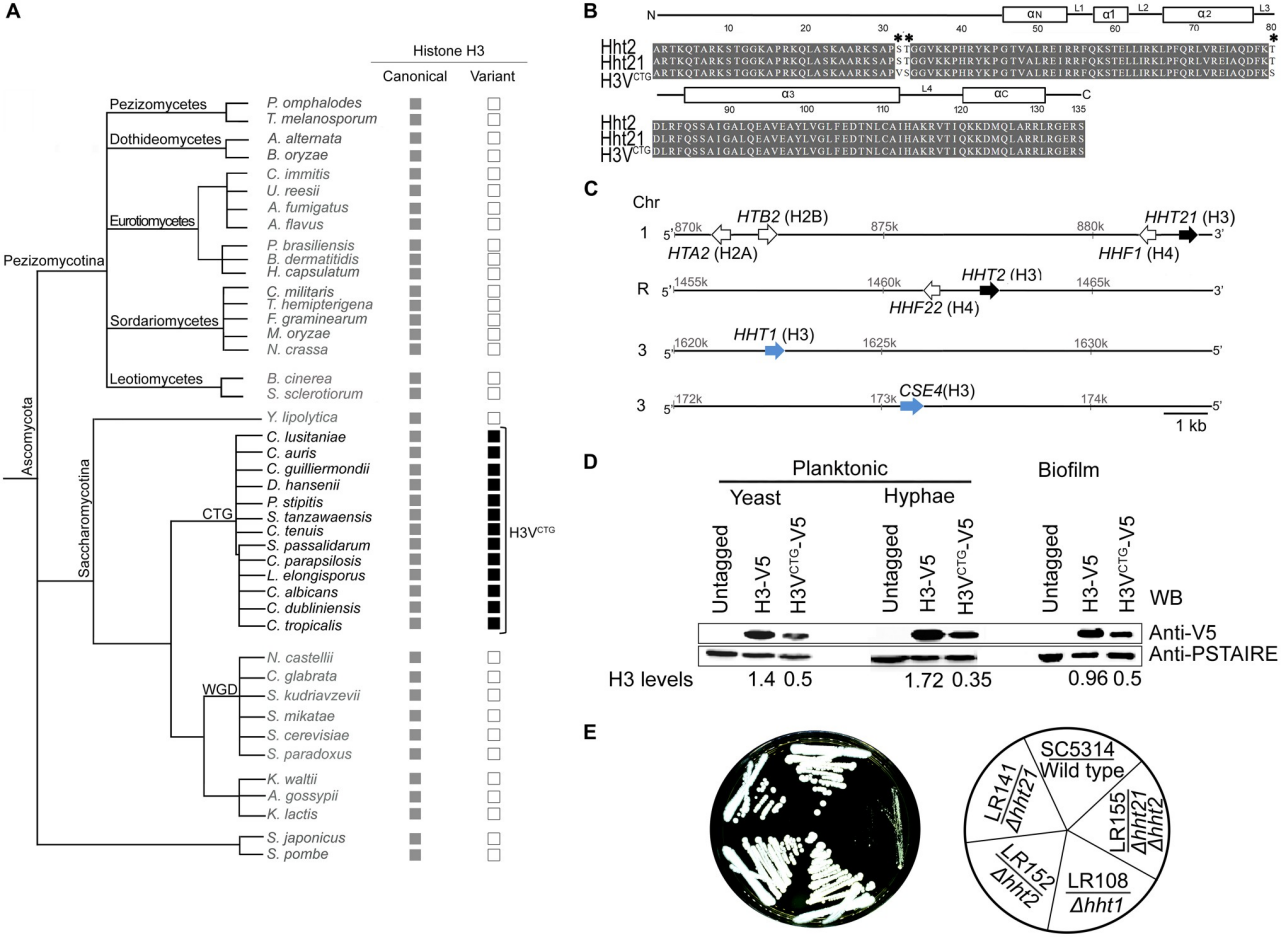
H3V^CTG^ is a CTG-clade specific histone H3 variant. (A) Phylogeny of fungi in the phylum Ascomycota analyzed in this study. The canonical histone H3 genes (gray box) along with the presence (black box) or absence (empty box) of the variant histone H3 of the corresponding species are shown. This tree is illustrative as the branches are not drawn to scale. The species included in this phylogeny are *Aspergillus fumigatus*, *A*. *flavus*, *Ashbya gossypii*, *Alternaria alternate*, *Botrytis cinerea*, *Blastomyces dermatitidis*, *Bipolaris oryzae*, *Candida lusitaniae*, *C*. *guilliermondii*, *C*. *tenuis*, *C*. *parapsilosis*, *C*. *albicans*, *C*. *dubliniensis*, *C*. *tropicalis*, *C*. *auris*, *C*. *glabrata*, *Coccidioides immitis*, *Cordyceps militaris*, *Debaryomyces hansenii*, *Fusarium graminearum*, *Histoplasma capsulatum*, *Kluyveromyces waltii*, *K*. *lactis*, *Lodderomyces elongisporus*, *Magnaporthe oryzae*, *Neurospora crassa*, *Naumovozyma castellii*, *Paracoccidioides brasiliensis*, *Pichia stipitis*, *Pyronema omphalodes*, *Spathaspora passalidarum*, *Suhomyces tanzawaensis*, *Saccharomyces kudriavzevii*, *S*. *makatea*, *S*. *cerevisiae*, *S*. *paradoxus*, *Schizosaccharomyces pombe*, *S*. *japonicus*, *Sclerotinia sclerotiorum*, *Torrubiella hemipterigena*, *Tuber melanosporum*, *Uncinocarpus reesii*, and *Yarrowia lipolytica*. (B) Amino acid sequence alignment of histone H3 proteins coded by *HHT2*, *HHT21*, and *HHT1* in *C*. *albicans*. Changes in the amino acid sequence among these histone H3 variants are marked by an asterisk. Identical amino acids are shaded, and numbers above the sequence denote amino acid locations on the primary protein sequence. Secondary structures such as α helices and loops of the corresponding sequence are indicated above the alignment. (C) Sketch showing locations of histone H3 genes and histone gene clusters on various chromosomes of *C*. *albicans*. (D) Expression levels of *C*. *albicans* histone H3 proteins encoded by *HHT21-V5* (H3-V5) and *HHT1-V5* (H3V^CTG^-V5) were monitored by western blot analysis in the planktonic (yeast and hyphae) and biofilm growth conditions. Histone H3 molecular weight is approximately 17 kDa. The parental strain SN148 was used as the untagged control, whereas PSTAIRE (approximately 34 kDa) was used as the loading control. (E) Wild-type SC5314 (*HHT1*/*HHT1*), variant histone H3 null mutant LR108 (*hht1*/*hht1*), canonical histone H3 mutants LR142 (*hht21/hht21*) and LR152 (*hht2/hht2*), and canonical histone H3 null strain LR155 (*hht21/hht21 hht2/hht2*) were streaked on YPD plates and grown at 30 °C for 3 days. Chr, chromosome; YPD, yeast peptone dextrose.

Using the *S*. *cerevisiae* histone H3 (YNL031C) amino acid sequence as the query in a basic local alignment search tool (BLAST) search, a variant of the core histone H3 protein, exclusively present in a group of ascomycetes belonging to the CTG clade (Fig 1A) was identified. The sequence of the newly identified CTG-clade-specific histone H3 variant is different from the core canonical histone H3, as well as the variant histone H3 present in Basidiomycota and Zoopagomycota fungal phyla. In *C*. *albicans*, *HHT2* (*ORF 19*.*1853*) and *HHT21* (*ORF 19*.*1061*) code for an identical polypeptide, the canonical histone H3, whereas *HHT1* (*ORF 19*.*6791*) encodes a variant protein differing at positions 31, 32, and 80 in the amino acid sequence when compared with *HHT2*/*HHT21* (Fig 1B and S1A Fig). These changes are found to be conserved in the variant histone H3 of most CTG-clade species (S1B Fig). Moreover, variations in the nucleotide sequences of *HHT1*, *HHT2*, and *HHT21* were inspected using genome sequence data available for 182 *C*. *albicans* isolates [35]. Only 2 synonymous SNPs were identified in *HHT1*, indicating that the amino acid sequence of Hht1 protein is invariant across the 182 tested *C*. *albicans* strains. Four synonymous and 1 nonsynonymous SNPs were found in *HHT2*, whereas 8 synonymous and 2 nonsynonymous SNPs were identified in *HHT21* (S1 Table). Notably, these variations do not occur at the 3 positions where Hht1 differs from Hht2 or Hht21 in the primary amino acid sequence. The presence of serine (S) and threonine (T) at position 31 and 80, respectively, in the canonical histone H3 (*HHT2* and *HHT21*) is similar to that of the mammalian histone H3.3. In addition, these 2 amino acid positions (31st and 80th) of the histone H3 polypeptide are known to tolerate variations as evident in other organisms [36]. Therefore, we propose that *HHT1* is indeed encoding a variant histone H3, possibly independently evolved in the CTG clade, and we named it as H3V^CTG^.

### Variant histone H3 is less abundant than the canonical histone H3 in major morphological forms of *C*. *albicans*

The canonical histone H3 genes *HHT2* and *HHT21* in *C*. *albicans* are divergently transcribed from the histone H4 genes, *HHF22* (*ORF 19*.*1059*) and *HHF1* (*ORF 19*.*1854*), respectively, whereas the variant histone H3 gene, *HHT1*, is located outside the canonical histone gene clusters (Fig 1C). Based on genomic locations and sequence features, we considered the polypeptide coded by *HHT21* or *HHT2* as the canonical histone H3 and the one coded by *HHT1* as the variant histone H3 (H3V^CTG^) found exclusively in the CTG-clade species. Reverse transcription PCR (RT-PCR) confirmed that both the canonical histone H3 and the variant histone H3 genes are transcribed (S2A and S2B Fig). Further, epitope tagging of these genes confirmed that the corresponding proteins are translated in planktonic (yeast and hypha) as well as biofilm grown cells of *C*. *albicans* (Fig 1D). Although no significant difference in relative abundance of the 2 canonical histone H3 proteins was observed in *C*. *albicans* (S2C Fig), variant histone H3V^CTG^ was expressed in lower abundance than the canonical histone H3 proteins in both planktonic and biofilm mode of growth in *C*. *albicans* (Fig 1D). Finally, indirect immunolocalization assays confirmed nuclear localization of both forms of histone H3 proteins, canonical H3 (LR143), or H3V^CTG^ (LR144) (S2D Fig). Although the canonical histone H3 localization was found to be uniform across the entire nucleus, a distinct scattered pattern of nuclear localization of variant histone H3 was observed.

### Variant histone H3 assembles into nucleosomes and can support *C*. *albicans* growth in the absence of canonical histone H3

To test the essentiality of H3V^CTG^ for viability of *C*. *albicans*, both alleles of *HHT1* were replaced with a recyclable knock-out *SAT1*-flipper cassette [37] in the wild-type *C*. *albicans* strain SC5314 (S3A and S3B Fig). H3V^CTG^ was found to be dispensable for survival of *C*. *albicans* in rich media because the homozygous null mutants lacking H3V^CTG^ (LR107 and LR108) and the parent wild-type strain grew at a similar rate (Fig 1E and S3C Fig). Similarly, each of the canonical histone H3 genes, *HHT2* or *HHT21*, was deleted individually by the *SAT1*-flipper cassette. Null mutants of either *HHT2* (LR152) or *HHT21* (LR141) did not show any significant growth defects (Fig 1E). We also examined the expression levels of variant histone H3 (Hht1-V5) in the absence of *HHT21* but did not observe any significant differences in the expression levels of Hht1 (S3D Fig).

To determine whether *C*. *albicans* could survive in the absence of canonical histone H3, all 4 alleles encoding the canonical histone H3 were knocked out by clustered regularly interspaced short palindromic repeats–CRISPR associated protein 9 (CRISPR-Cas9) [38, 39]. Null mutant cells (*hht2/hht2 hht21/hht21*) lacking the canonical histone H3 genes *HHT2* and *HHT21* (LR155) were viable but grew significantly slower compared with any of the single mutants of histone genes or wild type (Fig 1E). We also examined the cell morphology of canonical histone H3 null mutant. These cells showed an elongated cell morphology compared with the wild type, suggesting they are stressed [40, 41] (S3E Fig). These results suggest that variant histone H3 (*HHT1*) can partially fulfill the functions of canonical histone H3 and is thus probably assembled into nucleosomes to support growth of *C*. *albicans* in the absence of canonical histone H3 genes.

### Variant histone H3 acts as a determinant of major growth transitions in *C*. *albicans*

**Fig 2:**
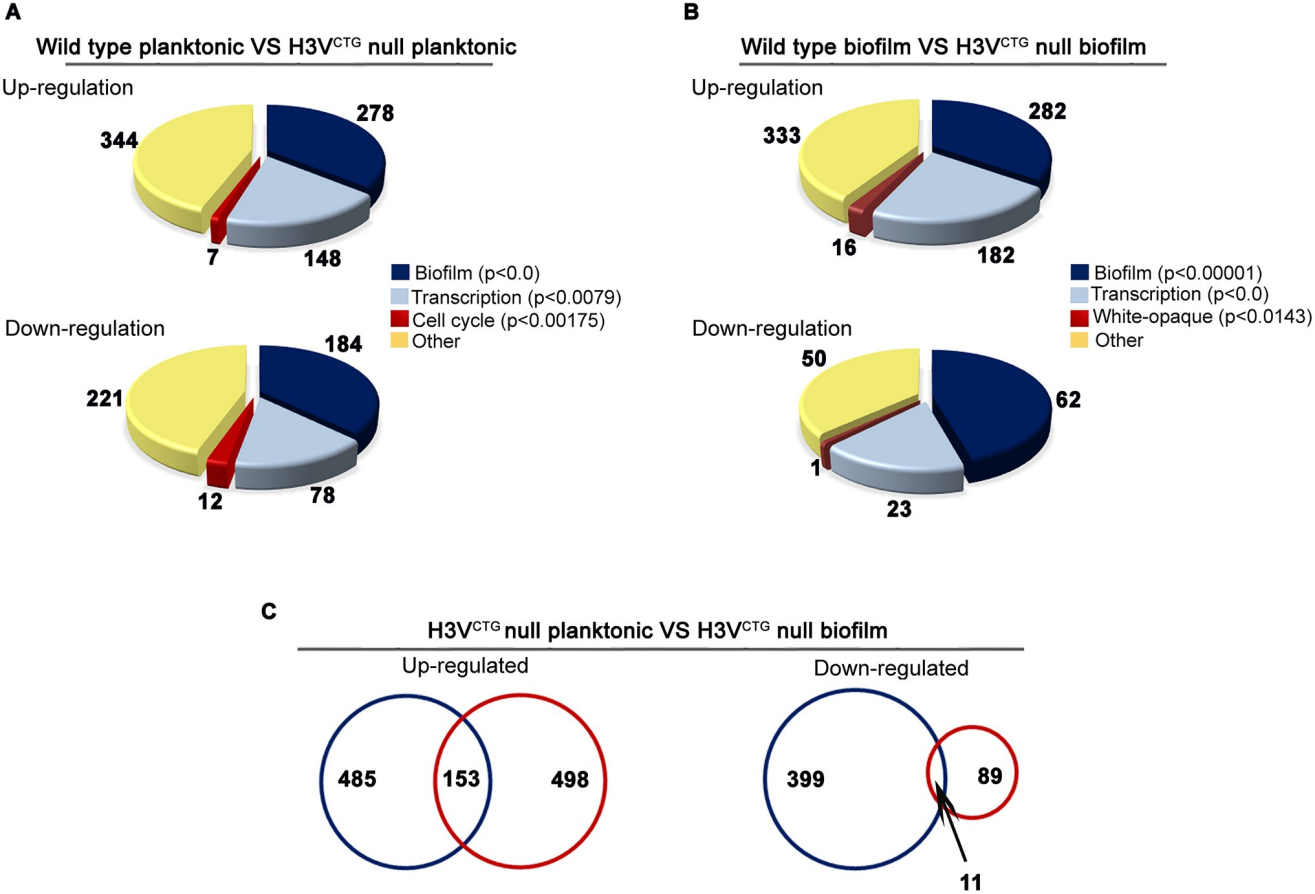
Loss of variant histone H3 activates the gene circuitry for biofilm development in planktonic cells. (A) Global gene expression array analysis was performed in the wild-type and null mutants for H3V^CTG^ grown in YPDU (planktonic mode). Functional classification of genome-wide expression data indicates that the most significantly altered pathways in the H3V^CTG^ null mutants are biofilm, transcription, and cell cycle. (B) A similar pattern of gene expression was observed when the indicated strains were grown in Spider medium under conditions that favor biofilm growth. (C) Genome-wide expression data were compared for common genes that are altered both in planktonic and biofilm growth conditions (blue and red circles, respectively) in the H3V^CTG^ null mutants and represented as Venn diagrams. A total of 153 up-regulated and 11 down-regulated genes are common between these 2 data sets. YPDU, yeast peptone dextrose supplemented with uridine.

To understand the biological relevance of H3V^CTG^ that is present only among the CTG-clade species of Ascomycota, a genome-wide transcriptome analysis of the H3V^CTG^ null mutant (*hht1/hht1*) was carried out. The gene expression profiles of 2 biological replicates of the H3V^CTG^ mutant strain (*hht1/hht1*), LR107 and LR108, as well as 2 different colonies of parental strain SC5314 (*HHT1/HHT1*) grown in planktonic conditions (yeast peptone dextrose supplemented with uridine [YPDU] broth at 30 °C) were analyzed. Approximately one-fifth of all *C*. *albicans* genes (1,048 genes) were found to have an altered expression (fold change > 1.5, *p* < 0.05) when H3V^CTG^ was absent (S1A Data). Out of these altered genes, 638 genes were up-regulated, whereas the remaining 410 genes were down-regulated in null mutants of H3V^CTG^ compared with the wild type (S1B and S1C Data). Functional categorization of up- and down-regulated genes suggested that the biofilm gene circuit was the most significantly altered pathway due to H3V^CTG^ deletion (Fig 2A and S4A Fig). Transcription and cell cycle were the other 2 significantly altered pathways in null mutants of H3V^CTG^ (*p* < 0.05; S1D Data). Transcription levels of a majority of biofilm-induced genes were found to be up-regulated, whereas biofilm-repressed genes were down-regulated when cells lacked H3V^CTG^. Adhesins and GPI-anchored cell wall proteins, known to play a crucial role during the biofilm development, were also up-regulated in H3V^CTG^ null mutants. In addition, *SAP5* and *SAP6* genes, which are shown to be involved in biofilm formation [42], were up-regulated in the H3V^CTG^ mutant. Based on the transcription profiling analysis, we posit that H3V^CTG^ favors planktonic growth over biofilm growth in *C*. *albicans*.

In the laboratory, *C*. *albicans* cells form biofilms under a variety of conditions on several substrates and media, including Spider medium [24, 27]. Altered expression of biofilm genes in the null mutant of H3V^CTG^ in the planktonic mode prompted us to perform a similar genome-wide transcriptome analysis after growing 2 independent colonies of wild-type (SC5314) and 2 biological replicates of H3V^CTG^ null mutant (LR107 and LR108) in Spider medium–induced biofilm conditions. In the biofilm-inducing conditions, 751 genes were found to be differentially expressed (fold change > 1.5, *p* < 0.05) between the wild-type and H3V^CTG^ null mutants (S1E Data). Among them, 651 genes were up-regulated, whereas the remaining 100 genes were down-regulated in the H3V^CTG^ null mutants (S1F and S1G Data) compared with the wild type (Fig 2B and S4B Fig). Again, the biofilm was the most significantly altered pathway (*p* < 0.05) between the null mutants of H3V^CTG^ and the wild type when the cells were grown in conditions that favor biofilm growth (Fig 2B and S1H Data).

As observed in the planktonic expression profile, several adhesins, GPI-linked cell wall proteins, and biofilm-induced genes were further up-regulated, whereas biofilm-repressed genes were further down-regulated in H3V^CTG^ null mutants compared with the wild type in the biofilm-induced condition. Among those with an altered expression, 164 genes were common to both planktonic and Spider biofilm transcriptome data sets (Fig 2C). Subsequently, we compared the altered gene-sets in the H3V^CTG^ mutants grown either in the planktonic (S4C and S4E Fig) or biofilm (S4D and S4F Fig) condition with genes known to be expressed differentially in planktonic and biofilm modes of growth [33] and represented in the form of a heat map and Venn diagram. These analyses revealed a significant overlap in the altered gene expression profile in the H3V^CTG^ mutants with the biofilm-specific genes.

### Loss of variant histone H3 induces biofilm gene circuitry during planktonic growth in vitro

**Fig 3:**
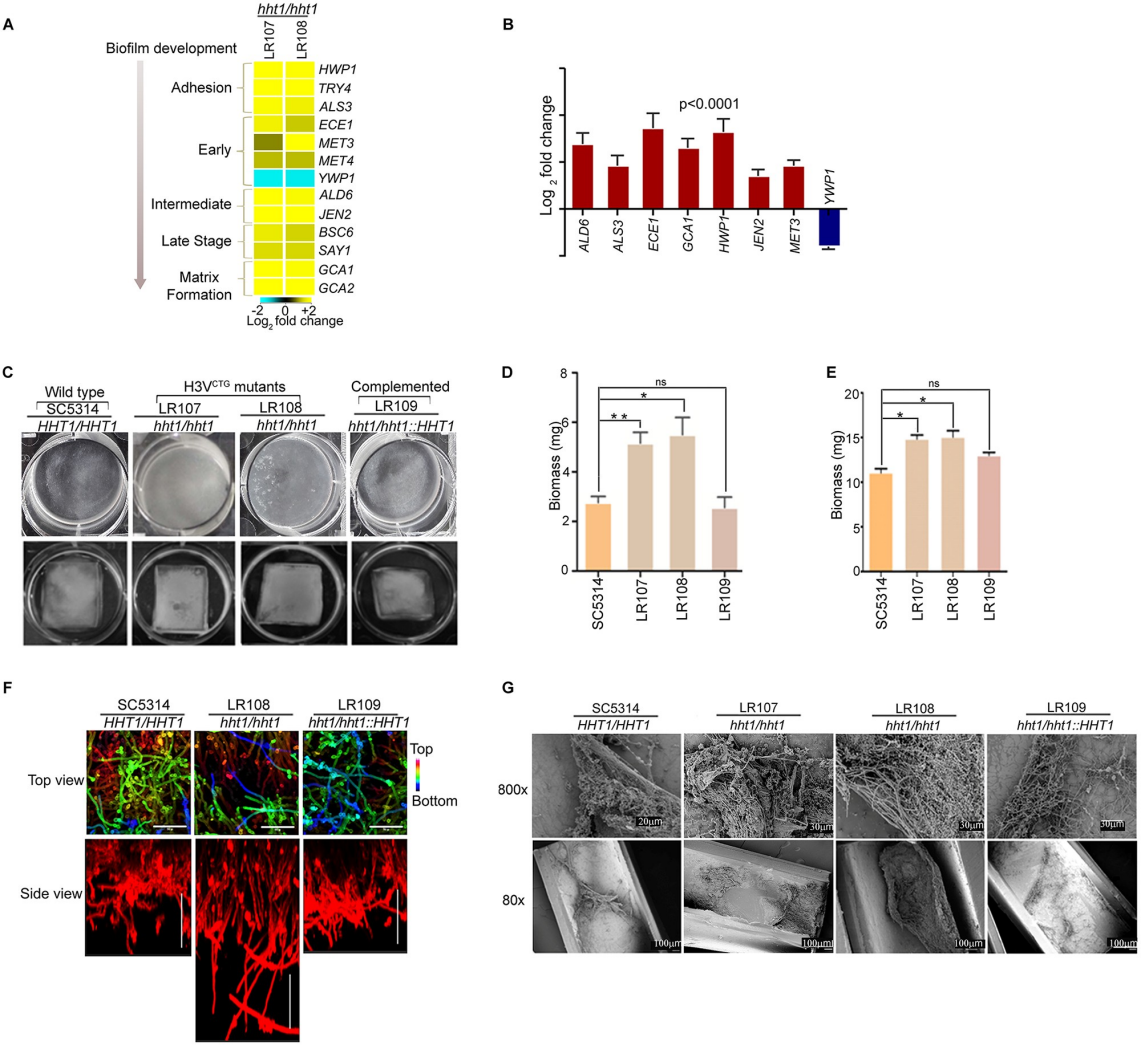
Variant histone H3 null mutants form more robust biofilm than wild type. (A) A heat map was generated for biofilm-related genes with altered levels of expression in the H3V^CTG^ null mutants compared with wild type. Yellow color represents up-regulation, whereas blue color represents down-regulation. Affected genes are arranged according to the step of biofilm development they are involved in. The arrowhead indicates the direction of maturation of biofilm. (B) qPCR analysis was performed for biofilm-related genes with wild-type and H3V^CTG^ null cells grown in YPDU under planktonic conditions. ΔCt values were derived after normalization of expression of biofilm genes with that of actin, whereas ΔΔCt values were calculated for relative expression of biofilm-related genes in the H3V^CTG^ null mutants compared with the wild type. The data underlying this figure can be found in S2 Data. (C) Biofilms were grown using the wild type (SC5314), 2 independent transformants of H3V^CTG^ null mutant (LR107 and LR108), and the complemented strain (LR109) in 6-well polystyrene plates in Spider medium at 37 °C. Biofilms were allowed to form for 24 hours at 37 °C (upper panel) or on silicone squares in YPDU medium for 24 hours at 30 °C (lower panel). The wells were washed to remove the nonadherent cells and photographed. (D) Biomass dry weights of the wild-type, H3V^CTG^ null mutants, and H3V^CTG^ complemented strains grown in Spider media at 37 °C are shown. The data underlying this figure can be found in S2 Data. (E) Biomass dry weights of the wild-type, H3V^CTG^ null mutant, and H3V^CTG^ complemented strains after growth in YPDU for 24 hours at 30 °C are shown. The data underlying this figure can be found in S2 Data. (F) Wild-type, H3V^CTG^ null mutant, and the H3V^CTG^ complemented strain were adhered to silicone squares in a 12-well polystyrene plate in YPD medium at 37 °C. Biofilms were allowed to form for 48 hours. Biofilms were stained with concanavalin A-Alexa 594 and imaged by CLSM. Images are projections of the top and side views. False color depth views were constructed, in which the blue color represents cells the closest to the silicone (bottom of the biofilm) and the red color represents cells the farthest from the silicone (top of the biofilm). Representative images of at least 3 replicates are shown. Scale bars: 50 μm. (G) The biofilm assay was performed in vivo using the rat catheter model. Strains were inoculated in the rat intravenous catheter and were allowed to form biofilms. After 24 hours of incubation, biofilms were visualized using SEM. The images are 80× and 800× magnification views of the catheter lumens. CLSM, confocal laser scanning microscopy; ns, not significant; qPCR, quantitative PCR; SEM, scanning electron microscopy; YPD, yeast peptone dextrose; YPDU, yeast peptone dextrose supplemented with uridine; ΔCt, difference in Ct values of a gene of interest and normalization control (in this case, actin); ΔΔCt, difference in Ct (threshold cycle) values of a gene of interest in a mutant strain compared to the wild type.

Analysis of the transcriptome data revealed that gene expression profiles previously linked to biofilm formation were enhanced in the H3V^CTG^ mutants compared with the wild type both in planktonic and biofilm conditions. Altered expression of a subset of critical biofilm-related genes (Fig 3A) was verified by quantitative PCR (qPCR) analysis (Fig 3B). Interestingly, *YWP1*, a biofilm-repressed gene, was down-regulated in H3V^CTG^ null mutants. Thus, the microarray data together with qPCR analysis established that H3V^CTG^ contributes to the maintenance of the planktonic growth by repressing the biofilm growth promoting gene circuitry when cells are grown in planktonic conditions. This finding led us to investigate the biofilm-forming ability of the null mutant of H3V^CTG^ compared with the wild type. To test this, wild-type SC5314 (*HHT1/HHT1*), H3V^CTG^ mutants LR107 and LR108 (*hht1/hht1*), and the H3V^CTG^ complemented strain LR109 (*hht1*/*hht1*::*HHT1*) were allowed to form biofilm in 6-well polystyrene plates in the Spider medium at 37 °C (Fig 3C, upper panel). A significant enhancement in biofilm growth of H3V^CTG^ mutants compared with the wild-type and H3V^CTG^ complemented strains was observed (Fig 3D). Together the results obtained thus far suggest that the gene circuit for biofilm development is activated in the H3V^CTG^ mutants even when these cells are grown in planktonic conditions. To verify this possibility, we performed the biofilm assay at 30 °C in YPDU medium on silicone squares; biofilm formation was found to be enhanced in H3V^CTG^ null mutants compared with the wild-type or the H3V^CTG^ complemented strain (Fig 3C lower panel and 3E), confirming that deletion of H3V^CTG^ promotes biofilm formation in YPDU medium at 30 °C.

In order to determine the thickness of biofilms formed by the *hht1* null mutant strain compared with wild type, confocal laser scanning microscopy (CLSM) was performed in vitro on silicone squares grown biofilms (see Materials and methods). Our CLSM results suggested that the *hht1* null mutant strain formed a thicker biofilm compared with either the wild-type or the complemented strain (Fig 3F). Finally, to examine the ultrastructure of biofilms formed by the wild-type and H3V^CTG^ null mutant, in vitro biofilms were allowed to grow on human urinary catheters for 48 hours (see Materials and methods). The catheter luminal surfaces were visualized by scanning electron microscopy (SEM). No significant differences were observed in the H3V^CTG^ null mutant compared with the wild type (S5 Fig).

### Absence of variant histone H3 enhances in vivo biofilm growth as well

Because of the presence of many uncharacterized host factors, in vivo biofilms may substantially differ from those formed in vitro. To examine whether absence of H3V^CTG^ enhanced biofilm formation in vivo, a well-established rat venous catheter model [43] was used. We inoculated catheters intraluminally with wild type (SC5314), H3V^CTG^ null mutants LR107 and LR108 (*hht1/hht1*), or H3V^CTG^ complemented strain LR109 (*hht1*/*hht1*::*HHT1*) of *C*. *albicans*. After 24 hours of biofilm growth, the catheters were removed, and catheter luminal surfaces were imaged by SEM (Fig 3G). Similar to in vitro results, significantly thicker biofilms were formed by the H3V^CTG^ null mutants (*hht1/hht1*) compared with the wild-type strain SC5314 or the H3V^CTG^ complemented strain LR109. Therefore, these in vivo as well as in vitro observations together support the conclusion that deletion of H3V^CTG^ results in the formation of more robust biofilms than those formed by wild-type *C*. *albicans*.

### Variant histone H3 acts as a molecular switch that favors planktonic growth over biofilm growth

**Fig 4:**
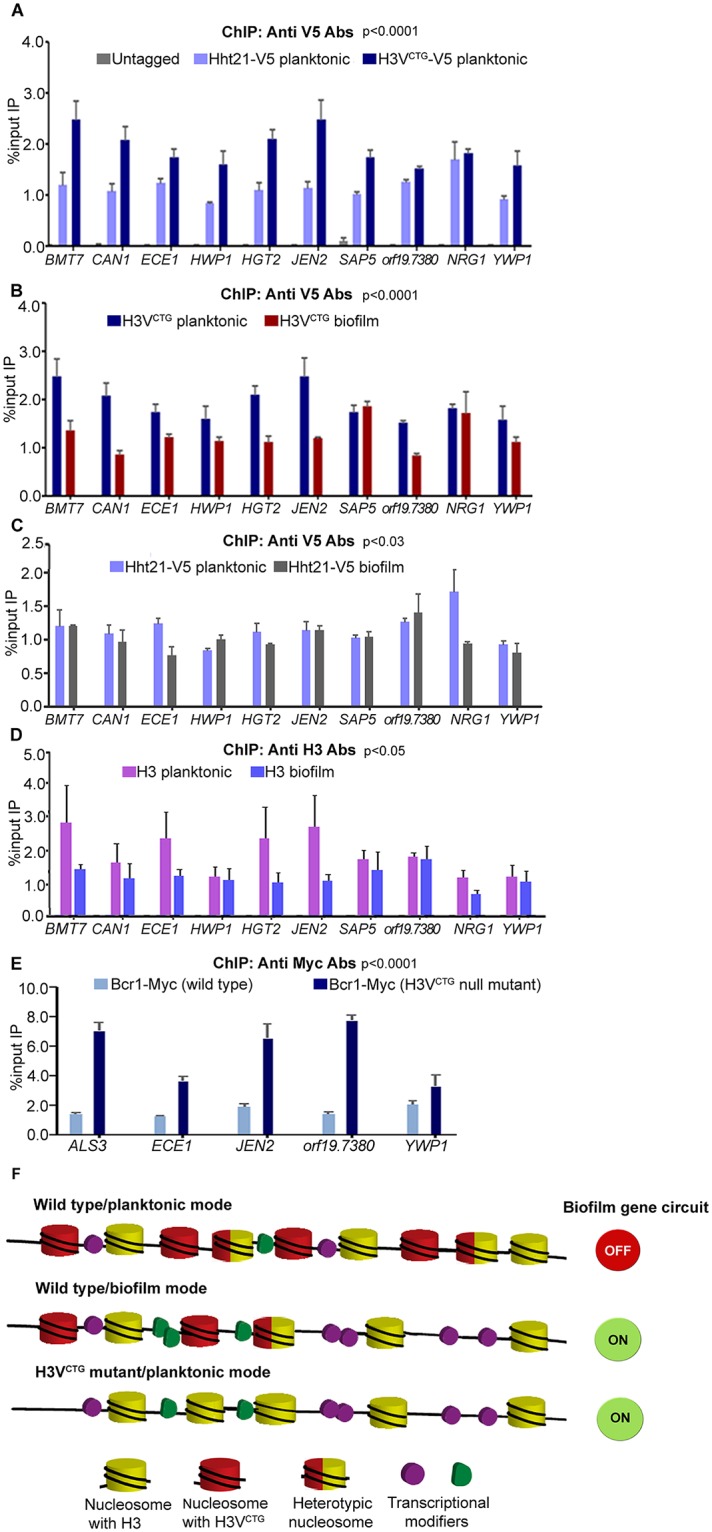
Variant histone H3 limits access of transcription modulators to promoters of biofilm-related genes. (A) ChIP assays with anti-V5 antibodies were performed in cells of LR143 (*HHT21/HHT21-V5*) and LR144 (*HHT1/HHT1-V5*) expressing a V5-tagged canonical histone H3 or variant histone H3 grown in planktonic conditions. IP DNA fractions were analyzed by qPCR with gene-specific promoter primer pairs (see S3 Table) for binding of either canonical histone H3 or H3V^CTG^. Quantitative PCR (qPCR) was also performed with untagged strain to detect the background DNA elution in the ChIP assay. The enrichment of canonical histone H3 or H3V^CTG^ to the promoters of biofilm-related genes is represented as a normalized percent input IP with SEM. The values from 3 independent ChIP experiments were plotted. A two-way ANOVA test was performed to determine statistical significance. The data underlying this figure can be found in S2 Data. (B) Similarly, ChIP assays with anti-V5 antibodies were performed in LR144 cells grown as a biofilm. The enrichment of H3V^CTG^ to the promoters of biofilm genes was compared in both planktonic and biofilm conditions. The data underlying this figure can be found in S2 Data. (C) ChIP assays with anti-V5 antibodies were performed in LR143 cells grown in biofilm conditions. The enrichment of H3-V5 to the promoters of biofilm genes was compared in both planktonic and biofilm conditions. The data underlying this figure can be found in S2 Data. (D) ChIP assays with anti-H3 antibodies were performed in SN148 cells grown in planktonic and biofilm conditions. The enrichment of H3 to the promoters of biofilm-related genes was compared in both planktonic and biofilm conditions. The data underlying this figure can be found in S2 Data. (E) The extent of binding of a biofilm master regulator, namely, Bcr1-myc, was examined either in presence or in absence of H3V^CTG^ in the planktonic mode of growth. Bcr1-myc ChIP assays were performed with anti-myc antibodies in both the parental strain (CJN1785) and the H3V^CTG^ mutant (LR133) expressing Bcr1-myc. IP DNA fractions were analyzed by qPCR with promoter specific primer pairs for the binding of Bcr1-myc. The enrichment of Bcr1-myc was calculated and normalized with *ORF19*.*874*. A two-way ANOVA test was performed to determine statistical significance. The data underlying this figure can be found in S2 Data. (F) A proposed model to depict the role of the H3V^CTG^ in creating repressive chromatin for the biofilm genes. In this model, during planktonic growth, H3V^CTG^ is more efficiently bound to the promoters of biofilm genes compared with the canonical H3 (Hht21-V5), although they can form heterotypic nucleosomes. Possibly H3V^CTG^ restricts binding of biofilm regulators to the promoters of target genes in the planktonic state, resulting in a tight regulation of their expression when cells grow in planktonic conditions. However, in the biofilm state, when nucleosomes containing the variant histone H3 are reduced, or in the absence of H3V^CTG^, an open chromatin structure is established that allows a more efficient access of biofilm regulators to the promoters of these genes. This causes either up-regulation of biofilm-induced genes (by transcription inducers) or down-regulation (by transcription repressors) of biofilm-repressed genes. Bcr1, biofilm and cell wall regulator 1; ChIP, chromatin immunoprecipitation; IP, immunoprecipitated; qPCR, quantitative PCR; RT-qPCR, real time quantitative PCR; SEM, standard error of the mean.

Enhancement of both in vitro and in vivo biofilm formation in the H3V^CTG^ mutant compared with the wild type motivated us to investigate the mechanism by which H3V^CTG^ negatively regulates biofilm growth in *C*. *albicans*. To understand this mechanism at the molecular level, the V5 epitope was used to tag either the variant histone H3 (H3V^CTG^) or the canonical histone H3 (encoded by *HHT21* as described above). Based on the complementation assay, the V5-tagged H3V^CTG^ (LR145 and LR146) was proved to be functional when expressed as the only copy in the cell (S6A Fig). Chromatin immunoprecipitation–quantitative PCR (ChIP-qPCR) analysis was performed either in the planktonic or biofilm growth conditions to determine the binding of both canonical and variant histone H3 to the promoters of a set of biofilm-induced and biofilm-repressed genes. The promoter of *ORF19*.*874*, which was unaltered in the genome-wide study above as well as in the previous report [33] during the planktonic to biofilm growth transition, was used to normalize the binding efficiency of histone H3 molecules in all ChIP-qPCR studies. The untagged parent strain (SN148) was used as a control to calculate the background enrichment. The promoters of biofilm-induced genes (*BMT7*, *CAN1*, *ECE1*, *HWP1*, *HGT2*, *JEN2*, and *SAP5*), biofilm-repressed genes (*NRG1* and *YWP1*), and also of an uncharacterized gene (*ORF19*.*7380*), to which 5 of the 6 biofilm master regulators bind, were examined to study the occupancy of either canonical (Hht21-V5) or variant histone H3 (H3V^CTG^-V5). The occupancy of H3V^CTG^ was significantly higher at the promoters of biofilm-related genes compared with that of the canonical histone H3 when the cells were grown in the planktonic mode of growth (Fig 4A and S1I Data). Therefore, we posit that a higher occupancy of the variant histone H3 at the promoters of biofilm genes may prevent access of gene expression modulators (transcription activators or repressors) required for induction of the biofilm gene circuitry under planktonic growth conditions. Further, the occupancy of the H3V^CTG^ was compared in both planktonic and biofilm growth conditions. A significant drop in the binding of H3V^CTG^ to the promoters of biofilm genes was observed during the transition from planktonic to biofilm growth, except at *SAP5* and *NRG1* promoters (Fig 4B). We also examined the occupancy of variant histone H3 to the gene body of biofilm-related genes in cells grown in planktonic and biofilm conditions. Similar to the promoter regions, we also observed a drop in the level of variant histone H3 in biofilm condition compared with the planktonic condition (S6B Fig). Further, we did not observe a similar trend of changes in the occupancy of canonical H3 to the promoters of biofilm genes between planktonic and biofilm conditions (Fig 4C). These analyses reveal that the binding of H3V^CTG^ to the promoter of each of the biofilm genes was more efficient in the planktonic than in the biofilm condition. Micrococcal nuclease (MNase) digestion was performed to determine the occupancy of these 2 histone H3 on the *BMT7* gene. Both total DNA MNase digestion and ChIP results show that both histone H3 can occupy the same regions (S6C Fig). This also suggests the possibility of formation of heterotypic nucleosomes, which will be addressed in future studies. Moreover, we examined the total pool of histone H3 molecules bound to the promoters of biofilm genes in both planktonic and biofilm growth by ChIP assays using antihistone H3 antibodies. Our results show a drop in the binding of total histone H3 to the promoters of *BMT7*, *CAN1*, *ECE1*, *HGT12*, *JEN1*, *SAP5*, and *NRG1* in biofilm conditions (Fig 4D). To assess the occupancy of one of the biofilm master regulators, namely, Bcr1, to the promoters of biofilm-related genes, we performed ChIP-qPCR analysis in the wild-type or the H3V^CTG^ null mutant expressing Bcr1-myc. Binding of Bcr1-myc was analyzed on promoters of a set of genes that showed an altered (up-regulation or down- regulation) expression in the H3V^CTG^ mutant by ChIP-qPCR (Fig 4E). The results of this analysis indeed confirmed that absence of H3V^CTG^ enhances the binding of the biofilm master regulator Bcr1 to the promoters of these genes in the planktonic conditions. These results were in accordance with the gene expression profile further strengthening the fact that H3V^CTG^ indeed plays a major role in repressing the biofilm promoting gene network during planktonic growth in *C*. *albicans*. Taken together with all the results presented so far, we propose a general model (Fig 4F) that suggests H3V^CTG^ is involved in modulating chromatin in a way that limits access of biofilm gene-specific transcription factors to the promoters of biofilm-related genes.

### Loss of variant histone H3 rescues biofilm-forming defects of mutants lacking master biofilm regulators

**Fig 5:**
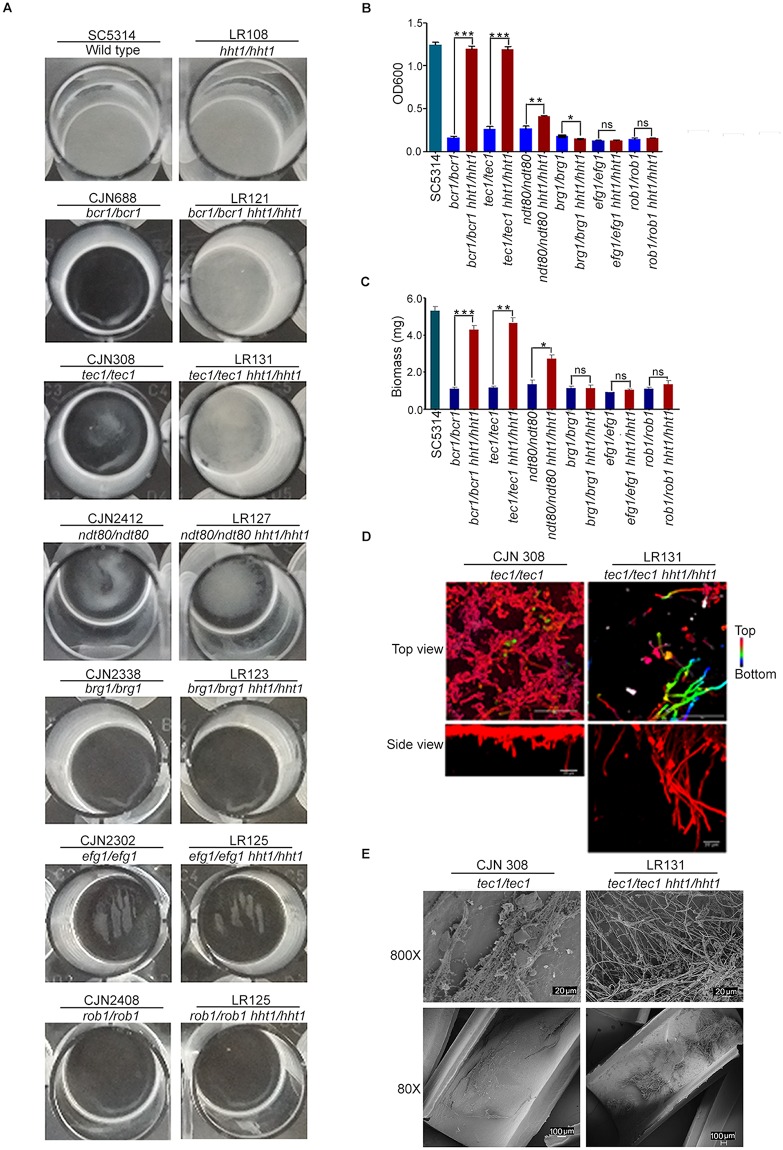
Deletion of the variant histone H3 rescues defects in biofilm formation associated with mutants of 3 biofilm master regulators. (A) In vitro biofilm formation assay was performed using single mutants of each of the 6 master regulators of biofilm formation, (Bcr1, Tec1, Ndt80, Brg1, Efg1, and Rob1) and the corresponding double-mutant strains in which H3V^CTG^ was deleted in each mutant background. Biofilms were grown in YPD medium in 24-well polystyrene plates for 24 hours at 37 °C. Biofilm formation defects were significantly rescued in *bcr1*, *tec1*, and *ndt80* null mutants in the absence of H3V^CTG^. (B) Biofilm formation was quantified using the Standard Optical Density Assay by measuring OD_600_ of the cells adhered to the bottom of the plates. In vitro biofilm assay was performed in YPD using indicated single- and corresponding double-mutant strains. Biofilms were grown in 24-well plates for 24 hours at 37 °C. Data are the mean of 3 independent wells per condition. Error bars represent the standard deviation. The data underlying this figure can be found in S2 Data. (C) Biomass of the wild type and each single- and double-mutant strains grown in YPD in 24-well plates for 24 hours at 37 °C. The data underlying this figure can be found in S2 Data. (D) *tec1* single mutant and *tec1 hht1* double mutant were adhered to silicone squares in a 12-well polystyrene plate in YPD at 37 °C, and biofilms were allowed to form for 48 hours at 37 °C. Biofilms were stained with concanavalin A-Alexa Fluor 594 and imaged by CLSM. Images represent projections of the top and side views. Representative images of at least 3 replicates are shown. Scale bars: 50 μm. (E) Biofilm assay was performed in vivo by using a rat catheter model. CJN308 (*tec1/tec1*) or LR131 (*tec1/tec1 hht1/hht1*) were inoculated in the rat intravenous catheter and were allowed to form a biofilm. After 24 hours of incubation, biofilms were visualized using SEM. The images are 80× and 800× magnification views of the catheter lumens. Bcr1, biofilm and cell wall regulator 1; Brg1, biofilm regulator 1; CLSM, confocal laser scanning microscopy; Efg1, enhanced filamentous growth protein 1; Ndt80, non-dityrosine 80; OD, optical density; Rob1, regulator of biofilm 1; SEM, scanning electron microscopy; Tec1, transposon enhancement control 1; YPD, yeast peptone dextrose.

Previously, it has been shown that the overexpression of *ALS3* in the *bcr1/bcr1* mutant rescues the defects in biofilm formation [44]. On the other hand, overexpression of Bcr1 target genes, such as *ALS1*, *ECE1* or *HWP1*, partially restores biofilm formation of the *bcr1/bcr1* mutant. Because several biofilm-related target genes, including *ALS3*, *ECE1* and *HWP1*, were up-regulated in the H3V^CTG^ null mutants and H3V^CTG^ binds to the promoters of many biofilm-related genes possibly to inhibit biofilm growth and to promote planktonic growth, we next examined the extent of biofilm formation in the absence of both a biofilm master regulator and the possible biofilm repressor H3V^CTG^. Deletion of each of the 6 master regulators, namely, *BCR1*, *BRG1*, *EFG1*, *NDT80*, *ROB1*, and *TEC1*, has been shown to severely hamper biofilm formation [33]. To test whether the deletion of H3V^CTG^ can rescue biofilm defects associated with the absence of biofilm master regulators, we deleted both copies of *HHT1* (H3V^CTG^) in each of the following mutant strains—*bcr1/bcr1*, *brg1/brg1*, *efg1/efg1*, *ndt80/ndt80*, *rob1/rob1*, and *tec1/tec1*—and examined the biofilm-forming ability of these double mutants by growing biofilms in YPD medium. Strikingly, biofilm growth was found to be significantly enhanced in the *bcr1*/*bcr1 hht1*/*hht1* and *tec1*/*tec1 hht1*/*hht1* double-mutant strains and also partially in *ndt80/ndt80 hht1/hht1* compared with *bcr1*/*bcr1*, *tec1*/*tec1*, or *ndt80/ndt80*, respectively (Fig 5A). The remaining 3 double mutants (*brg1*/*brg1 hht1*/*hht1*, *efg1*/*efg1 hht1*/*hht1*, and *rob1*/*rob1 hht1*/*hht1*) did not show any significant change in biofilm formation compared with the mutants lacking only the master regulator (Fig 5A). Next, the enhancement of biofilm formation observed in these double-mutant strains was quantified by standard optical density measurement as well as dry biomass of biofilm formed (Fig 5B and 5C). These results strongly suggest that variant histone H3 is a major regulator of biofilm formation in *C*. *albicans*. We measured by CLSM the thickness of the biofilm formed by the *tec1 hht1* double-mutant on silicone squares compared to the *tec1* mutant alone (Fig 5D). Finally, we examined in vivo biofilm formation of *tec1/tec1* as well as *tec1/tec1 hht1/hht1* mutant using the rat catheter model (Fig 5E). The double-mutant strain *tec1/tec1 hht1/hht1* formed enhanced biofilms compared with those formed by the *tec1/tec1* single mutant in both in vitro and in vivo conditions. Our results thus confirm that in the absence of H3V^CTG^, biofilm defects in the *tec1/tec1* mutant can be rescued both in vitro and in vivo. We speculate that the genes that are regulated by Bcr1, Ndt80, and Tec1 are derepressed at the chromatin level in the absence of H3V^CTG^. These results together confirm that H3V^CTG^ negatively regulates biofilm growth in *C*. *albicans*.

### Co-occurrence of amino acid residues at position 31 and 32 is essential for the function of variant histone H3

**Fig 6:**
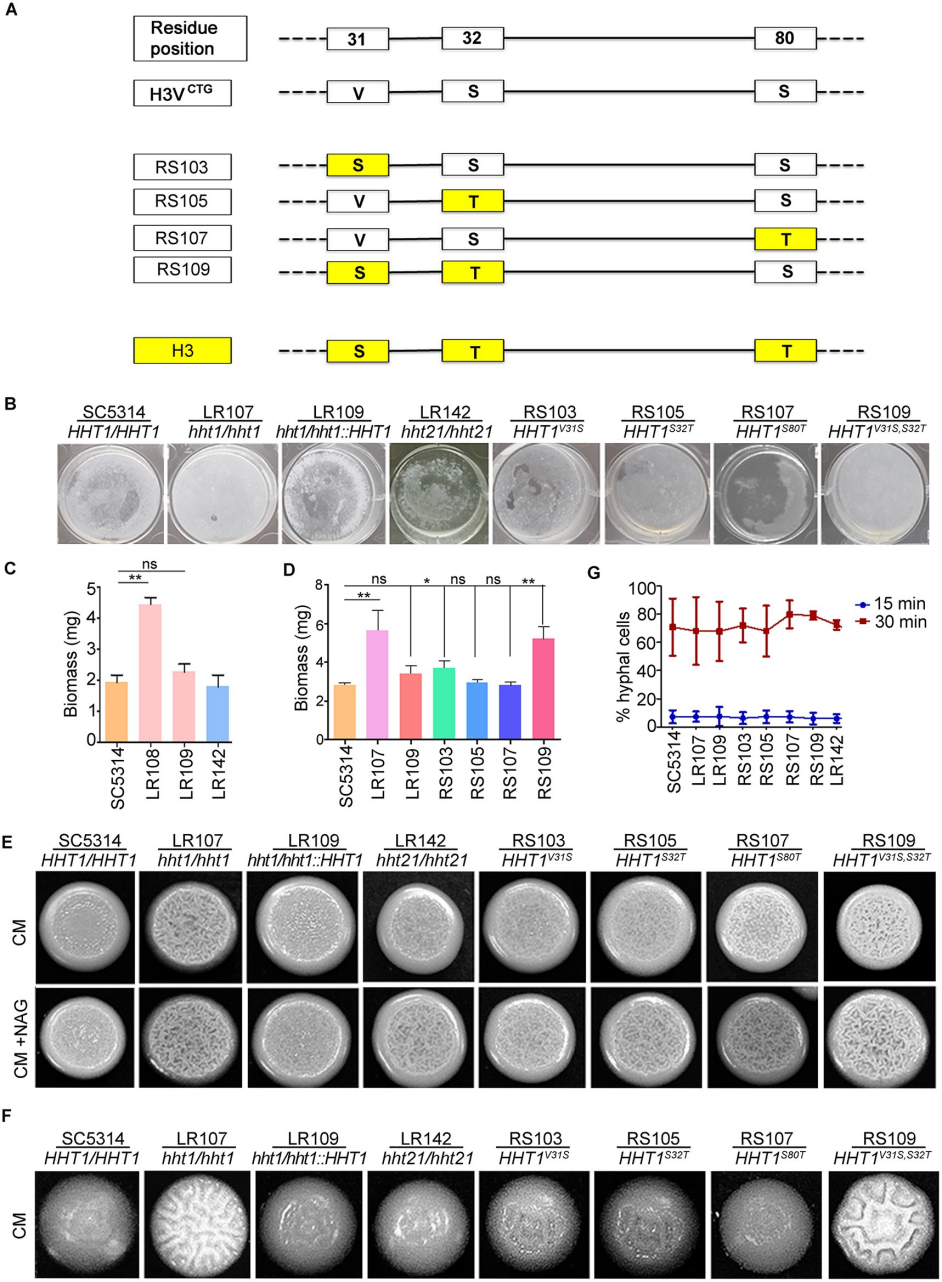
Amino acid residues 31 and 32 are essential for the function of H3V^CTG^. (A) Schematics represent various point-mutant strains constructed by changing amino acid residues at positions 31, 32, and/or 80 of variant histone H3 to that of the canonical histone H3. (B) SC5314, H3V^CTG^ null mutant LR108, H3V^CTG^ complemented strain LR109, canonical histone H3 mutant LR142 (*hht21/hht21*), point-mutant strains at position 31 (RS103), 32 (RS105), 80 (RS107), and point-mutant strain at positions 31 and 32 (RS109) were allowed to form biofilms in YPDU for 48 hours at 37 °C; wells were washed to remove the nonadherent cells and photographed. (C) Biomass dry weights of the wild-type, H3V^CTG^ null mutant, H3V^CTG^ complemented, and *hht21/hht21* strains grown in YPDU at 37 °C. The data underlying this figure can be found in S2 Data. (D) Biomass dry weights of the wild-type, H3V^CTG^ null mutant, H3V^CTG^ complemented, and point-mutant strains grown in YPDU at 37 °C. The data underlying this figure can be found in S2 Data. (E) SC5314, H3V^CTG^ null mutant LR108, H3V^CTG^ complemented strain LR109, canonical histone H3 mutant LR142 (*hht21/hht21*), point-mutant strain at position 31 (RS103), 32 (RS105), 80 (RS107), and point-mutant strain at position 31 and 32 (RS109) were spotted on synthetic dextrose medium complemented with essential amino acids (CM) agar plates, and CM+NAG (synthetic dextrose with 1 mM N-acetyl glucosamine) agar plates, and incubated for 3 days at 37 °C. (F) The extent of filamentation was monitored for the indicated strains by growing colonies from single cells on CM medium at 37 °C. (G) SC5314, H3V^CTG^ H3 null mutant LR107, H3V^CTG^ complemented strain LR109, point-mutant strains at position 31 (RS103), 32 (RS105), 80 (RS107), point-mutant strain at positions 31 and 32 (RS109), and canonical histone H3 mutant LR142 (*hht21/hht21*) were allowed to form filaments in liquid YPD with 10% FBS at 37 °C. The proportion of hyphal cells formed by these strains are plotted. The data underlying this figure can be found in S2 Data. CM, complete media; CM+NAG, complete media supplemented with N-acetyl glucosamine; FBS, fetal bovine serum; ns, not significant; YPDU, yeast peptone dextrose supplemented with uridine.

We were also interested to know the contribution of each of the 3 amino acids residues that are different between variant and canonical histone H3. To understand this, we generated single-point mutant strains of Hht1 at each of the positions 31, 32, and 80 similar to that of the canonical histone H3 sequence (Fig 6A). In addition, we were curious to know whether the enhancement in biofilm formation was specifically associated with the loss of the variant histone H3 or if it could be due to the loss of overall histone H3 levels in the cell. We examined the extent of biofilm formation in the null mutant of *HHT21* as well and did not observe any increase in biofilm formation. (Fig 6B and 6C). This confirms that the enhancement of biofilm growth is specifically associated with the loss of H3V^CTG^. In addition, we did not observe any significant differences in biofilm formation between wild-type and point-mutant strains (Fig 6B and 6D). We also generated a double mutant at positions 31 and 32 of Hht1 to change the amino acid residues similar to those of the canonical histone H3 at the corresponding positions. The double mutant yielded a phenotype similar to that of the null mutants of variant histone H3 (Fig 6B and 6D). This suggests that co-occurrence of amino acid residues at positions 31 and 32 is essential for variant histone H3 function as a biofilm repressor during planktonic growth. This further confirms that the phenotype observed in null mutants of variant histone H3 is specifically associated with variant histone H3 and not due to the global levels of histone H3.

### Variant histone H3 null mutant is hyperfilamentous

To test whether variant histone H3 has an effect on filamentation, we performed filamentation assays with wild-type SC5314 (*HHT1/HHT1*), H3V^CTG^ (*hht1/hht1*) mutants LR107 and LR108, and the H3V^CTG^ complemented strain LR109 (*hht1*/*hht1*::*HHT1*) on solid media. Colony wrinkling was enhanced in the mutant strains (*hht1/hht1*) compared with the wild-type (*HHT1*/*HHT1*) and the H3V^CTG^ complemented strain LR109 (*hht1*/*hht1*::*HHT1*) at both 30 °C and 37 °C (S7A Fig). This hyperfilamentation phenotype was specific to H3V^CTG^ mutants because null mutants of neither canonical histone H3 genes produced this phenotype (Fig 6E and S7B Fig). We also examined the extent of filamentation in the point-mutant strains. Our results confirm that the double mutant at positions 31 and 32 yields a phenotype similar to that of the null mutant of variant histone H3 (Fig 6E). The extent of filamentation was also found to be enhanced at the level of colonies derived from a single cell (Fig 6F and S7C Fig). Further, to test whether the increase in filamentation observed during growth on solid media can be found in liquid culture, hyphal growth was triggered in YPD containing fetal bovine serum at 37 °C. We did not observe any significant differences in the filamentation patterns between the wild type, the *hht1/hht1* mutant strains, the H3V^CTG^ complemented strain LR109 (*hht1*/*hht1*::*HHT1*), and the *hht21/hht21* null mutant or point-mutant strains (Fig 6G).

Finally, we performed a comparative transcriptome analysis of filamentation-specific genes between the wild type in filamentation inducing conditions [45] and H3V^CTG^ null mutant in planktonic and biofilm conditions. Our analysis does not show a significant overlap of genes with altered expression between *hht1* null mutants and existing filamentation-specific expression profile (S7D Fig). Taken together, our results suggest that the enhancement in filamentation leading to a more robust biofilm formation in the H3V^CTG^ null mutants is only associated with the solid surface media.

## Discussion

**Fig 7:**
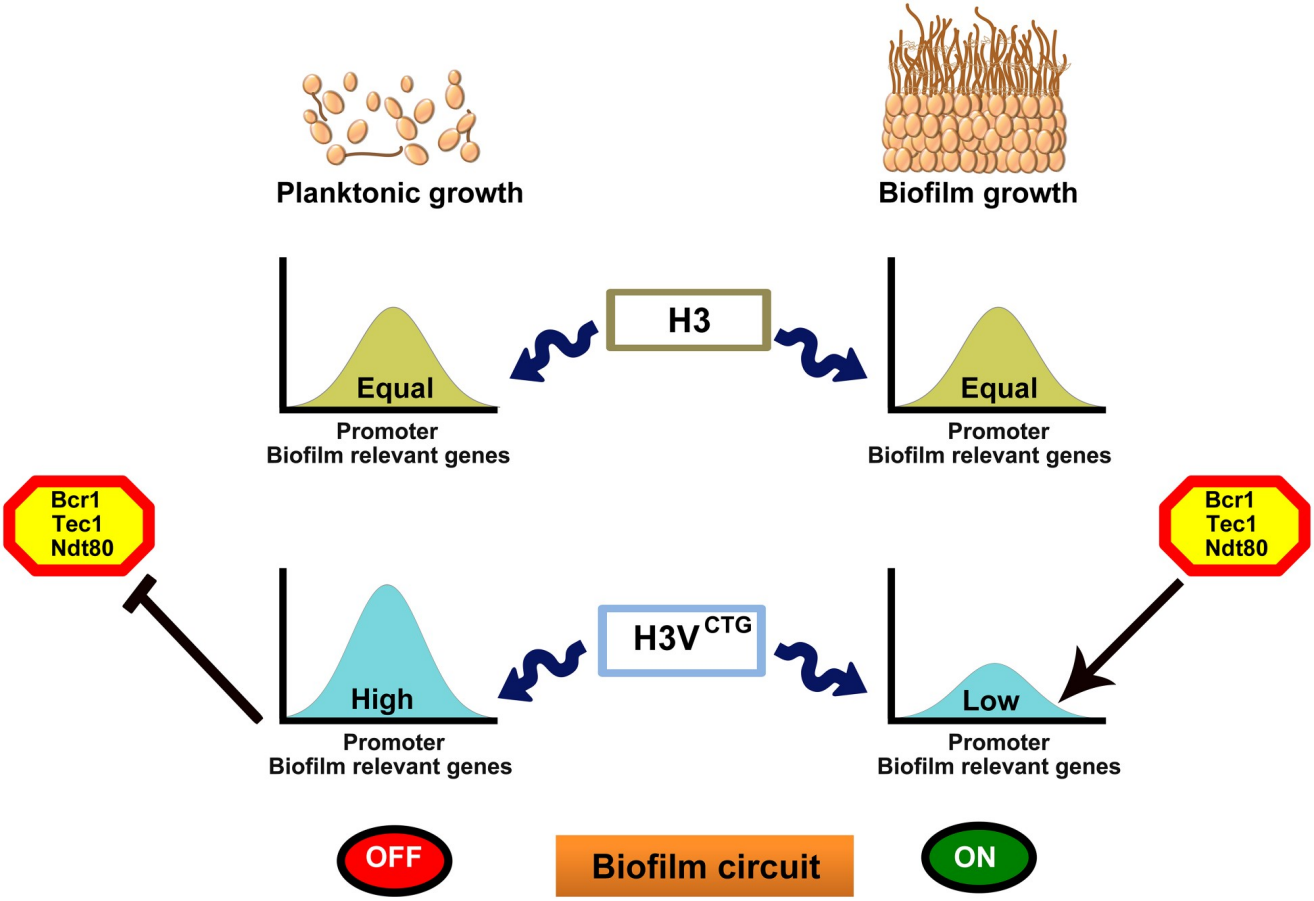
The CTG-clade–specific histone H3 evolved as the molecular switch of morphological growth transitions in *C*. *albicans*. The variant histone H3 (H3V^CTG^) is less abundant than its canonical form in *C*. *albicans*. *C*. *albicans* can either grow as free-floating individual cells in the planktonic mode in a flask or as a three-dimensional community on a biotic or abiotic surface to form biofilms. Relative occupancy of the canonical histone H3 remains unaltered on the promoters of several biofilm-related genes tested in planktonic and biofilm cells. On the other hand, occupancy of the variant histone H3, H3V^CTG^, is significantly higher on the promoters of the same set of biofilm genes in the planktonic cells compared with those grown in the biofilm conditions. Based on experimental evidence, we posit that the variant histone H3 nucleosomes make biofilm gene promoters less permissive for binding of transcription modulators (transcription activators or repressors) of biofilm-related genes. When H3V^CTG^ levels drop, biofilm transcription modulators bind to the promoters to modulate the genetic circuitry to favor the biofilm mode of growth. Thus, we unravel a new mechanism modulated at the chromatin level by a CTG-clade–specific histone H3 variant to balance a medically relevant growth transition of a major human pathogen. Bcr1, biofilm and cell wall regulator 1; Ndt80, non-dityrosine 80; Tec1, transposon enhancement control 1.

*C*. *albicans* is an opportunistic fungal pathogen capable of forming biofilms both in vitro and in vivo. Although the transcriptional regulation during the formation of biofilms has been studied, the molecular mechanisms at the chromatin level during this process are less understood. In this study, we identified a variant histone H3 that is exclusively present in the CTG-clade species, including *C*. *albicans*, but absent in all other ascomycetous fungi analyzed. The histone H3 variant, H3V^CTG^, is not essential for survival of *C*. *albicans*, but the null H3V^CTG^ mutant exhibits a more robust biofilm growth as well as enhanced filamentation on solid surfaces compared with the wild type. Supported by a series of evidence, we believe that the CTG-clade specific H3V^CTG^ might have evolved to make chromatin less accessible for biofilm transcription modulators to favor the yeast and planktonic growth of *C*. *albicans* (Fig 7). First, ChIP-qPCR analysis revealed that the occupancy of H3V^CTG^ is higher at the promoters of biofilm-related genes compared with that of the canonical histone H3 in planktonic cells. Second, the occupancy of H3V^CTG^ is reduced at the promoters of biofilm-specific genes during the transition of *C*. *albicans* cells from planktonic to biofilm growth mode. Third, the absence of H3V^CTG^ enhances the binding of Bcr1, a master regulator of biofilm formation, to the promoters of the biofilm-related genes. Finally, deletion of H3V^CTG^ rescues biofilm defects associated with 3 biofilm transcription factors, proposed as master regulators, of the biofilm gene circuitry. This repressive behavior of biofilm growth is associated specifically with H3V^CTG^, because alterations of the amino acid residues of Hht1 simultaneously at positions 31 and 32 to those of the canonical histone H3 yield phenotypes similar to those associated with the *hht1* null mutant.

Morphogenesis has been extensively studied as a potential pathogenic trait [46]. Most human fungal pathogens, including *C*. *albicans*, *Histoplasma capsulatum*, *Penicillium marneffei*, *Cryptococcus neoformans*, and *Coccidiodes immitis* are dimorphic or polymorphic in nature. *C*. *albicans* cells that are locked either in yeast (*efg1 cph1* double mutant) or in the filamentous form (*tup1* mutant) show compromised virulence [47, 48]. *C*. *albicans* cells tend to form hyphal filaments at 37 °C. The hyperfilamentation nature of the H3V^CTG^ mutant on solid surfaces even at 30 °C indicates that H3V^CTG^ suppresses surface-associated filamentation genes at low temperatures.

The genome-wide transcriptome analysis suggests that the pathway most significantly affected in the absence of H3V^CTG^ is the biofilm regulatory network. *C*. *albicans* is one of the very few fungal species that can form an efficient biofilm in a healthy mammalian host. The biofilm circuit in *C*. *albicans* probably evolved following divergence from closely related, nonpathogenic fungi [33]. One important step in the biofilm development is the yeast-to-hyphal transition with hyphal filaments being essential components of a mature biofilm. For example, the *bcr1* mutant is defective in biofilm formation, and it also fails to form hyphae under specific conditions, underscoring the importance of hyphal filaments in biofilm formation [49]. Bcr1 is required for expression of cell surface adherence genes (*ALS1*, *ALS3*, and *HWP1*) that are also induced during hyphal growth, suggesting that several common genes play a role during both biofilm and hyphal growth [27]. Genes, namely, *CAN2*, *HWP1*, and *TPO4*, are down-regulated in mutants of all 6 biofilm master regulators, whereas they are all up-regulated in the H3V^CTG^ null mutant during planktonic growth. This inverse correlation suggests that H3V^CTG^ plays an antagonistic role in the biofilm regulatory circuitry compared with that of previously identified biofilm master regulators [33]. Furthermore, we show that this circuit is not only derepressed in the planktonic condition but also in biofilm-induced conditions in the absence of H3V^CTG^. Taken together with these results, it is expected that the absence of H3V^CTG^ should enhance biofilm formation in *C*. *albicans*.

The most compelling evidence that H3V^CTG^ acts as a negative regulator of biofilm development in *C*. *albicans* came from an enhanced biofilm formation by H3V^CTG^ mutant cells both in vitro and in vivo conditions compared to the wild-type or the H3V^CTG^ complemented strain. Further, the H3V^CTG^ null mutant formed an enhanced biofilm in a condition that generally favors planktonic growth. This is probably due to the formation of filaments on solid surfaces by H3V^CTG^ mutant cells at low temperatures and the fact that biofilm and filamentation genes are derepressed at this restrictive temperature.

In metazoans, histone H3 variants play a key role in the differentiation of embryonic stem cells [50]. During cellular differentiation, stem cells undergo dramatic morphological and molecular changes through selective silencing and activation of lineage-specific genes. Therefore, it may be possible that similar to metazoans, H3V^CTG^ evolved to govern the morphogenetic switch in unicellular organisms such as yeast of the CTG clade. Along with various pathogenic traits, up-regulation of several adhesion factors in the absence of H3V^CTG^ indicates that the variant histone H3 appeared in the CTG-clade species to maintain the balance between commensal and pathogenic states. *C*. *albicans* is a parasite because it primarily exists in a host. Thus, we propose that H3V^CTG^ provides a balance on the pathogenic attributes of *C*. *albicans*, perhaps to enhance its success as a commensal. Studies are in progress to understand how chromatin changes modulated by this novel histone H3 variant bring genetic innovations to regulate one of the most dramatic morphological growth transitions in a medically relevant human fungal pathogen.

## Materials and methods

### Ethics statement

All animal procedures were approved by the Institutional Animal Care and Use Committee at the University of Wisconsin according to the guidelines of the Animal Welfare Act, the Institute of Laboratory Animal Resources Guide for the Care and Use of Laboratory Animals, and Public Health Service Policy under protocol MV1947. Ketamine and xyalazine were used for anesthesia. CO_2_ asphyxiation was used for euthanasia at the end of study.

### Media and growth conditions

*C*. *albicans* strains were grown in YPDU (1% yeast extract, 2% peptone, 2% dextrose, and 100 μg/ml uridine) at 30 °C unless stated otherwise. For biofilm formation, YPDU, Spider media (1% peptone, 1% yeast extract, 1% manitol, 0.5% NaCl, and 0.2% K_2_HPO_4_) at 37 °C [51] were used. The filamentation assays were performed with complete medium, Spider medium, and complete media containing 1 mM N-acetylglucosamine [52, 53]. Each strain was diluted to an OD_600_ of 0.080, spotted on a plate, and incubated for 2 or 3 days at 30 °C and photographed [52]. Serum (10%) was used for induction of hyphae in YPDU media at 37 °C. *C*. *albicans* cells were transformed by the standard lithium acetate method as described previously by Sanyal and colleagues [54]. Nourseothricin was used at a concentration of 100 μg/ml.

### Strain construction

All *C*. *albicans* strains and primer sequences used for the construction of strains in this study are listed in S2 and S3 Tables, respectively. Detailed information about the strain construction is available in the S1 Text.

### RNA extraction and cDNA synthesis

RNA was isolated from *C*. *albicans* strains with the mirVana RNA isolation kit (Ambion, AM1560). Cells were grown in YPDU to an OD_600_ = 0.5 or in biofilm condition from 48 hours grown biofilms. Approximately 4 × 10^7^ cells were taken for spheroplasting. Cells were pelleted down at 4,000 rpm, were washed with 1 ml of Y1 buffer (2.5 M sorbitol, 0.5 M EDTA [pH 8]) and finally were resuspended in 2 ml of Y1 buffer. Approximately 20 μl of lyticase and 2 μl of β-Mercaptoethanol were added and spheroplasting was done at 30 °C at 70 rpm. After 90% spheroplasting was achieved, spheroplasts were isolated by centrifugation at 1,800 rpm for 5 minutes, and RNA was isolated with the mirVana RNA kit. A total of 500 ng of purified RNA was used to make cDNA. The reaction mixture contained 4 μl of RNA, 4 μl of 5× RT buffer, 2 μl of 10 mM dNTPs, 2 μl of 0.1 M DTT, 2 μl of 10 pMol gene-specific reverse primer (H3-4), 1 μl of Superscript Reverse Transcriptase (Invitrogen, 18064–022) added in a final volume of 20 μl. Reactions were carried out at 37 °C for 60 minutes followed by heat inactivation at 85 °C for 5 minutes.

### qPCR

The *C*. *albicans* wild-type SC5314 and H3V^CTG^ null mutant strains were grown in YPD medium till OD = 0.5. RNA was isolated as described before treated with DNase I (NEB, M03032), and the quality of RNA was examined on agarose gel. To ensure the absence of genomic DNA, a control PCR reaction was performed before the reverse transcription step. cDNA was synthesized by reverse transcription using M-MuLV reverse transcriptase (Fermentas, EP0732) and Oligo(dT) primers (Sigma, O-4387). Primers designed for RT-PCR reactions were between 100 and 110 bp long (listed on S3 Table). Analysis of melting curves was also performed to ensure specific amplification without any secondary nonspecific amplicons (melting curve temperatures, used are 78°C (*ACT1*), 76 °C (*ALD6*), 79 °C (*ECE1*), 80.5 °C (*HWP1*), 78 °C (*GCA1*), and 79.5 °C (*YWP1*)). PCR was carried out in a final volume of 20 μl using iQSYBR Green supermix (BIO-RAD, 170-8880AP). The RT-PCR analysis was carried out in i-Cycler (BIO-RAD) using the following reaction conditions: 95 °C for 2 minutes, then 40 cycles of 95 °C for 30 seconds, 55° C for 30 seconds, 72 °C for 30 seconds. Fold difference in expression of mRNA was calculated by the ΔΔCt method (RT-PCR applications guide BIO-RAD) [55]. Actin was used as normalization control.

### Cell lysate and western blot analysis

Western blot analysis was performed as described before by Chatterjee and colleagues [56]. *C*. *albicans* strains were grown in YPDU till OD_600_ = 1. For hyphal induction, cells were grown in the presence of 10% serum, and to obtain biofilms, *C*. *albicans* was grown in 6-well polystyrene plates in YPDU. Cells were harvested, washed with lysis buffer (0.2 M Tris, 1 mM EDTA, 0.39 M (NH_4_)_2_SO_4_, 4.9 mM MgSO_4_, 20% glycerol, 0.95% acetic acid [pH 7.8]), resuspended in 0.5 ml of the same buffer, and disrupted using acid-washed glass beads (Sigma, G8772) by vortexing 5 minutes (1 minute vortexing followed by 1 minute cooling on ice) at 4 °C. *C*. *albicans* cell lysates were electrophoresed on a 12% SDS-PAGE gel and blotted onto a nitrocellulose membrane in a semidry apparatus (BIO-RAD). The blotted membranes were blocked with 5% skim milk containing 1× phosphate buffered saline (PBS; pH 7.4) for 1 hour at room temperature and then incubated with the following dilutions of primary antibodies: anti-V5 antibodies at a dilution of 1:5,000 (Invitrogen, R96025), anti-PSTAIRE antibodies (Abcam, ab10345) at a dilution 1:3,000. Anti-PSTAIRE antibodies recognize Cdc28 and are widely used as a protein loading control in *C*. *albicans*. Next, membranes were washed 3 times with PBST (0.1% Tween-20 in 1× PBS) solution. Anti-rabbit HRP conjugated antibodies (Bangalore Genei, 105499) and anti-mouse IgG-HRP antibodies (Bangalore Genei, 105502) were added at a dilution of 1: 1,000 and incubated for 1 hour at room temperature followed by 3 to 4 washes with PBST solution. Signals were detected using the chemiluminescence method (Super Signal West Pico Chemiluminescent substrate, Thermo scientific, 34080).

### Indirect immunofluorescence

Indirect immunofluorescence was performed as described before by Sanyal and Carbon and Chatterjee and colleagues [54, 56]. Asynchronously grown *C*. *albicans* cells were fixed with a one-tenth volume of formaldehyde (37%) for 15 minutes at room temperature. Antibodies used were diluted as follows: 1:100 for mouse anti-V5 antibodies (Invitrogen, R96025). The dilutions for secondary antibodies used were Alexa Fluor 488 goat anti-mouse IgG (Invitrogen, A-11001) diluted 1:1,000. DAPI (4, 6-Diamino-2-phenylindole) (Sigma, D9542) was used to stain the nuclei of the cells. Cells were examined under 100× magnifications using a GE Delta vision microscope. The digital images were processed with Adobe Photoshop.

### Gene expression design and data analysis

The concentration of the extracted RNA was evaluated using Bioanalyzer (Agilent), whereas purity of the extracted RNA was determined by the standard procedure for the same by measuring A_260_ and A_280_ using a Nanodrop Spectrophotometer (Thermo Scientific). The samples were labeled using Agilent Quick Amp labeling kit (Part number 5190–0442). A total of 500 ng RNA was reverse transcribed using Oligo(dT) primer tagged to the T7 promoter sequence. The cDNA obtained was converted to double stranded cDNA in the same reaction. Further, cDNA was converted to cRNA in the in vitro transcription step using T7 RNA polymerase, and Cy3 dye was added into the reaction mix. During cRNA synthesis, Cy3 dye was incorporated to the newly synthesized strands. cRNA obtained was cleaned up using Qiagen RNeasy columns (Qiagen, 74106). Concentration and the amount of dye incorporated in each sample were determined using a Nanodrop. Samples that passed the QC for the specific activity (minimum RNA concentration 500 ng/μl and absorbance 260/280 = 2) were taken for hybridization. A total of 600 ng of labeled cRNA was hybridized on the array (AMADID 29460) using the Gene Expression Hybridization kit (part number 5190–0404; Agilent) in Sure hybridization Chambers (Agilent) at 65 °C for 16 hours. Hybridized slides were washed using Agilent Gene Expression wash buffers (part no. 5188–5327). The hybridized, washed microarray slides were then scanned on a G 2600 D scanner (Agilent). Data extraction from images was done using Feature Extraction software version 10.7 of Agilent. Microarray data was preprocessed using Limma package (Smyth and colleagues, 2005) of statistical R language. Briefly, preprocessing includes (a) background correction (b) within array normalization and (c) fitting data to linear model. Finally, an empirical Bayes moderated statistics was applied to find significant changes in the expression levels. We used a cut-off of *p* < 0.05 and |fold change| > 1.5 for the differential expression of mutant over wild type to define differentially expressed genes. Differentially regulated genes were clustered using hierarchical clustering to identify significant gene expression patterns. All genes represented on the array are annotated using CGD database. The differentially regulated genes (up or down) were categorized under biofilm, cell cycle, transcription, and others based on the gene ontology annotations. Chi-square test was applied to know the significance of the categorized functions. Gene expression data have been deposited into the NCBI Gene Expression Omnibus (GEO) portal under the accession number GSE72824.

### In vitro biofilm growth and biomass determination

#### Biofilm growth in Spider media at 37 °C and dry biomass estimation

To perform the genome-wide expression analysis in the biofilm-induced condition, biofilms were grown in vitro in Spider medium by growing the biofilms directly on the bottom of 6-well polystyrene plates (CELLSSTAR, 657160). Biofilms were developed as described by Nobile and Mitchell [49]. Strains were grown in YPD overnight at 30 °C, diluted to an optical density at 600 nm (OD_600_) of 0.5 in 3 ml of Spider medium. The 6-well plates had been pretreated overnight with fetal bovine serum (FBS) and washed with 2 ml PBS. The inoculated plate was incubated at 37 °C for 90 minutes at 110 rpm agitation for initial adhesion of cells. Plates were then washed with 2 ml PBS, and 3 ml of fresh Spider medium was added, and plates were at 37 °C for 48 hours. Biofilm growth assays of the wild type, the H3V^CTG^ null mutants, and the *HHT1* complemented strain were performed by diluting ON culture to an OD_600_ of 0.2 in 3 ml Spider medium. To estimate the dry biomass of biofilms, biofilms were scrapped, and the content of each well was transferred to preweighed nitrocellulose filters. Biofilm-containing filters were dried overnight at 60 °C and weighed. The average total biomass for each strain was calculated from 3 independent samples after subtracting the mass of the empty filter.

### In vitro biofilm growth in YPD medium at 30 °C and biomass determination

To examine the biofilm formation ability of H3V^CTG^ mutant strains in YPDU medium at 30 °C, in vitro biofilm growth assays were carried out by growing biofilms on either the silicone squares substrate as previously described by Nobile and Mitchell [49]. Biofilms were developed as described before, except in this case, YPDU was the medium of choice and the temperature was 30 °C. Finally, medium was removed, and the silicone squares gently washed with 1× PBS prior to being photographed. For dry biomass measurements, the medium was removed and 2 ml of 1× PBS was added to each well to remove unadhered cells. Biofilms were then scrapped, and the content of each well was transferred to preweighed nitrocellulose filter paper, dried, and weighed as described before.

### CLSM for biofilm imaging

Biofilms were imaged as described before by Nobile and Mitchell [49]. Biofilms were grown on silicone squares for 48 hours, gently washed with 1× PBS, and stained with 50 μg ml^−1^ of concanavalin A-Alexa Fluor 594 (Invitrogen, C-11253) for 1 hour at 110 rpm. CLSM was performed at PPMS facility of Institut Pasteur using an upright LSM700 microscope equipped with a Zeiss 40X/ 1.0 W plan-Apochromat immersion objective. Silicone squares were placed in a petri dish, and the biofilms were covered with 1× PBS. Images were acquired and assembled into maximum intensity Z-stack projection using ZEN software (Zeiss).

### In vivo rat catheter biofilm model

To form biofilm in vivo, the rat central-venous catheter infection model [43] was used, as described previously Nobile and colleagues, Andes and colleagues, Nobile and colleagues, and Dalal and colleagues [33, 43, 44, 57]. For this specific pathogen free Sprague-Dawley rats weighing 400 g each were used. A heparinized (100 U/ml) polyethylene catheter with 0.76 mm inner and 1.52 mm outer diameters was inserted into the external jugular vein. The catheter was secured to the vein with the proximal end tunneled subcutaneously to the midscapular space and externalized through the skin. The catheters were inserted 24 hours prior to infection to permit a conditioning period for a deposition of host protein on the catheter surface. Infection was achieved by intraluminal instillation of 500 μl *C*. *albicans* cells (10^6^ cells/ml). After a 4 hour dwelling period, the catheter volume was withdrawn, and the catheter was flushed with heparinized 0.15 M NaCl. Catheters were removed after 24 hours of *C*. *albicans* infection to assay biofilm development on the intraluminal surface by SEM. Catheter segments were washed with 0.1 M phosphate buffer (pH 7.2) fixed in 1% glutaraldehyde/4% formaldehyde, washed again with phosphate buffer for 5 minutes, and placed in 1% osmium tetraoxide for 30 minutes. The samples were dehydrated in a series of 10 min ethanol washes (30%, 50%, 85%, 95%, and 100%) followed by critical point drying. Specimens were mounted on aluminium stubs, sputter coated with gold, and imaged using a Hitachi S-5700 or JEOL JSM-6100 SEM in the high-vacuum mode at 10kV. Images were processed using Adobe Photoshop software.

### ChIP

The ChIP assays were performed as described previously by Chatterjee and colleagues and Mitra and colleagues [56, 58]. Briefly, each strain was grown until exponential phase (approximately 2 × 10^7^ cells/ml) or grown in biofilm condition for 24 hours, and cells were cross-linked with 1% final concentration of formaldehyde for 13 minutes. Chromatin was isolated and sonicated to yield an average fragment size of 300 to 500 bp. The DNA was immunoprecipitated with anti-V5 antibodies (final concentration 20 μg/ml) or anti H3 antibodies (Abcam, ab1791) or anti myc antibodies (Cal Biochem, OP10) and purified. The total, immunoprecipitated (IP) DNA, and beads only material were used to determine the binding of H3V^CTG^ to the promoters of biofilm genes by qPCR, as described before. The template used was as follows: 1 μl of 1:100 dilution for input and 1 μl of 1:2 dilution for IP for H3-V5 ChIP and undiluted for H3 and myc ChIP. The conditions used in qPCR were as follows: 94 °C for 2 minutes; the 40 cycles of 94 °C for 30 seconds, 58 °C for 30 seconds, 72 °C for 45 seconds. The results were analyzed using CFX Manager Software. The graph was plotted according to the percentage input method [59], and the formula for calculation is 100 × 2^(adjusted *Ct* input − adjusted *Ct* of IP)^. Here, the adjusted value is the dilution factor (log2 of dilution factor) subtracted from the *C*_*t*_ value of diluted input/IP.

## Supporting information

S1 Fig Multiple sequence alignment of histone H3 variants in the CTG-clade species. (A) Comparative analysis of nucleotide sequences of *HHT2*, *HHT21*, and *HHT1*, the 3 *C*. *albicans* histone H3 encoding genes. (B) Comparative analysis of the amino acid sequences of histone H3 variants of the CTG-clade species. Amino acid sequences were aligned using the Bioedit software. Identical amino acids are indicated as dots and amino acids differing from *C*. *albicans* Hht1 are given as single letter symbol: *C*. *albicans* (Ca), *C*. *dubliniensis* (Cd), *C*. *tropicalis* (Ct), *C*. *parapsilosis* (Cp), *C*. *orthopsilosis* (Co), *Lodderomyces elongisporus* (Le), *Debaryomyces hansenii* (Dh), *Pichia stipitis* (Ps), *C*. *tenuis* (Cte), *Spathaspora passalidarum* (Sp), *C*. *guilliermondii* (Cg), *C*. *lusitaniae* (Cl). Hht1 is labeled as 1 (e.g., Ca1), whereas Hht2/Hht21 is labeled as 2 (e.g., Ca2) for each species.
(TIF)

S2 Fig Both canonical and variant histone H3 are expressed and localized in *C*. *albicans* nucleus. (A) Structure of the plasmids carrying *HHT21* or *HHT1* genomic DNA sequences as well as upstream and downstream sequences. *HHT21* locus was amplified with the primer pair 1061USFP and 1061DSRP (coordinates 881615 to 883328 of Chromosome 1), whereas the *HHT1* locus was amplified with the primer pair H3.6791USFP and H3.6791DSRP (coordinates 57523 to 59160 of Chromosome 3). PCR fragments were cloned into TZ57R/T (Thermo Scientific). Given PCR conditions were optimized to selectively amplify either *HHT21* or *HHT1*. (B) RT-PCR was performed with primers selectively amplifying either *HHT21* or *HHT1*. −RT acts as negative control, whereas actin serves as positive control. The 217 bp on +RT lanes represents the canonical/variant histone H3 (*HHT21* or *HHT1*) and the lower band of 110 bp on the same lanes corresponds to actin. (C) Expression levels of V5-tagged *C*. *albicans* canonical histone H3 proteins (approximately 17 kDa), namely, Hht2 and Hht21, were monitored by western blot analysis in the yeast form. The parental strain SN148 was used as the untagged control, whereas PSTAIRE (approximately 34 kDa) was used as the loading control. (D) Subcellular localization of the canonical histone H3 (Hht21-V5) or the variant form (Hht1-V5) was performed in the yeast form of *C*. *albicans* with anti-V5 antibodies. Nuclei were stained with DAPI. Co-localization of histone H3 with DNA was shown by merging the images. Both interphase (unbudded) and mitotic cells (large budded) are shown. Scale bars: 5 μm. RT-PCR, reverse transcription PCR.
(TIF)

S3 Fig Variant histone H3 can partially fulfill the function of canonical histone H3. (A) Structural schematic of the *HHT1* locus in the diploid *C*. *albicans* strains, namely, the wild-type SC5314 *(HHT1*/*HHT1*), heterozygous mutants (*HHT1*/*hht1*) LR103 and LR104, and homozygous null mutants LR105 and LR107 (*hht1*/*hht1*). Genomic NcoI sites are marked by down arrows. Location of the variant histone H3 gene, *HHT1*, is shown as a gray box, whereas the *SAT1* cassette is shown as a black box. (B) Isolated genomic DNA from each indicated strain was digested with NcoI and Southern hybridized with an upstream probe (shown by the black bar in panel A). Expected results for the correct transformants were obtained. (C) Growth assays were performed by growing SC5314, LR107, LR108, and the H3V^CTG^ complemented strain LR109 in YPDU liquid medium until the stationary phase was reached. Optical density was measured by using Varioskan Flash (Thermo Scientific). The data underlying this figure can be found in S2 Data. (D) Expression levels of histone H3 were monitored by western blot analysis of tagged strains LR144 (H3V^CTG^-V5) and LR149 (H3V^CTG^-V5 *hht21/hht21*) grown as yeast; the expected size of Hht1 is approximately 17 kDa. The parental strain SN148 was used as the untagged control, whereas PSTAIRE (approximately 34 kDa) was used as the loading control. Levels of histone H3 are normalized with the corresponding PSTAIRE levels, and values are indicated below each lane. (E) Wild-type (SC5314) and canonical histone H3 null mutant (LR155) strains were grown in YPD medium, and DIC images were taken at 60×. Scale bars: 2 μm. YPD, yeast peptone dextrose; YPDU, yeast peptone dextrose supplemented with uridine.
(TIF)

S4 Fig Expression data (≥1.5 fold difference with *p* ≤ 0.05) of genes from wild-type and H3V^CTG^ mutants are illustrated as heat-map. (A) Comparison of gene expression profiles of wild-type SC5314 and variant histone H3 null mutants (*hht1/hht1*) grown in planktonic condition. (B) Comparison of gene expression profiles of wild-type SC5314 and variant histone H3 null mutants (*hht1/hht1*) grown as biofilm. (C) Comparison of common gene expression profiles between *hht1/hht1* null mutant cells grown in planktonic condition and differentially expressed genes in biofilm mode from Nobile and colleagues [33]. (D) Similarly, common genes between *hht1/hht1* null mutant cells grown in biofilm condition and wild-type differentially expressed genes grown in biofilm conditions [33]. (E) Comparative analysis of altered biofilm-related genes between the H3V^CTG^ null mutants grown in planktonic conditions (blue circles) with differentially expressed genes in biofilm conditions (red circles; [33]) in the wild type. A total of 198 up-regulated and 70 down-regulated genes are common between these 2 data sets. (F) Similarly, differentially expressed genes in the H3V^CTG^ null mutants grown in biofilm-inducing conditions (blue circles) was compared with the previously published biofilm-induced microarray data of the wild type (red circles; [33]). A total of 93 up-regulated and 7 down-regulated genes are found to be common between these 2 data sets.
(TIF)

S5 Fig Ultrastructure of in vitro grown biofilms. (A)Wild-type SC5314 (*HHT1/HHT1*), H3V^CTG^ null mutant, LR108 (*hht1/hht1*), and the H3V^CTG^ complemented strain LR109 were allowed to form biofilms on human urinary catheters for 48 hours at 37 °C. The catheter luminal surfaces were visualized by SEM. SEM, scanning electron microscopy.
(TIF)

S6 Fig Binding of variant histone H3 to gene bodies in planktonic and biofilm conditions. (A) To examine the functionality of V5 epitope-tagged strains, wild-type, *hht1* null mutant and H3V^CTG^-V5/*hht1* strains were spotted on Spider medium. (B) ChIP assays with anti-V5 antibodies were performed in the strain LR144 expressing H3V^CTG^-V5 and grown in planktonic or biofilm conditions. The enrichment of H3V^CTG^-V5 to the gene bodies of biofilm genes was compared in both planktonic and biofilm conditions. The data underlying this figure can be found in S2 Data. (C) MNase digestion of the genomic DNA isolated from cells of the wild-type and *hht1/hht1* null mutant (LR107) grown in planktonic conditions was performed. The MNase digested DNA was precipitated and quantified by qPCR. The normalized Ct values represent the occupancy of nucleosomes at 2 previously known locations (NBR1, NBR2) at the promoter of the *BMT7* gene. These 2 regions have been predicted to be nucleosome bound. Similarly, MNase ChIP was performed in LR143 (Hht21-V5) and LR144 (H3V^CTG^-V5) strains. A greater enrichment of H3V^CTG^-V5 compared to Hht21-V5 was observed at those 2 regions (NBR1, NBR2). The data underlying this figure can be found in S2 Data. ChIP, chromatin immunoprecipitation; MNase, micrococcal nuclease; qPCR, quantitative PCR.
(TIF)

S7 Fig The variant histone H3 mutant strain is hyperfilamentous on solid surfaces at both 30° C and 37 °C. (A) SC5314, null mutants of H3V^CTG^ (LR107, LR108), and the H3V^CTG^ complemented strain LR109 (*hht1/hht1*::*HHT1*) were grown in liquid YPD medium and then spotted on CM and CM-containing N-acetyl glucosamine agar plates and incubated for 2 to 3 days at 30 °C. (B) SC5314, LR108, H3V^CTG^ complemented strain LR109 (*hht1/hht1*::*HHT1*), and canonical histone H3 mutant LR153 (*hht2/hht2*) were grown in liquid YPD and then spotted on plates containing the indicated media and incubated for 2 to 3 days at 37 °C. (C) Similarly, the extent of filamentation was monitored for the indicated strains by growing colonies from single cells on CM medium at 37 °C. (D) Comparative analysis of filamentation-specific genes after excluding genes common between biofilm and filamentation pathways. The Venn diagrams show the comparison of differentially expressed genes between wild-type SC5314 strain grown in filamentation-induced conditions with H3V^CTG^ null mutant grown either in planktonic or biofilm-inducing conditions. An arrow shows the overlapping genes. CM, complete media; YPD, yeast peptone dextrose.
(TIF)

S1 Text Construction of *C. albicans* strains, plasmids, and description of supplemental methods used in this study. (DOCX)

S1 Table Polymorphisms in the histone H3 gene among 182 *C. albicans* isolates. (DOCX)

S2 Table Strains and plasmids used in this study. (DOCX)

S3 Table Primers used in this study. (DOCX)

S1 Data Transcriptome profile of wild-type and *hht1* null mutants grown in planktonic and biofilm conditions. S1A Table contains a list of all transcriptionally altered genes in the null mutants of *hht1* as compared to the wild type during planktonic growth. S1B and S1C Table contain lists of up- and down-regulated genes in *hht1* null cells during planktonic growth, respectively. S1D Table contains p values of pathways altered in *hht1* null mutants when grown in the planktonic condition. S1E Table contains a list of all transcriptionally altered genes in the null mutants of *hht1* as compared to the wild type during biofilm growth. S1F and S1G Table contain lists of up- and down-regulated genes in *hht1* null cells in biofilm, respectively. S1H Table contains p values of pathways altered in *hht1* null mutants grown in the biofilm condition. S1I Table contains normalized percent immunoprecipitated values.
(XLSX)

S2 Data Data files related to Figs 3B, 3D, 4A, 4B, 4C, 4D, 4E, 5B, 5C, 6C and 6D, as well as S3C, S6B and S6C Figs. (XLSX)

## Abbreviations

Als: agglutinin-Like Sequence
Bcr1: biofilm and cell wall regulator 1
BLAST: basic local alignment search tool
Brg1: biofilm regulator 1
ChIP: chromatin immunoprecipitation
Chr: chromosome
CLSM: confocal laser scanning microscopy
CM: complete media
CM+NAG: complete media supplemented with N-acetyl glucosamine
CRISPR-Cas9: clustered regularly interspaced short palindromic repeats–CRISPR associated protein 9
Eap1: enhanced adherence to polystyrene 1
Efg1: enhanced filamentous growth protein 1
FBS: fetal bovine serum
Flo8: flocculation 8
Gal4: galactose metabolism 4
GEO: gene expression omnibus
GPI: glycosylphosphophatidylinositol
hH4v: histone H4 variant
Hwp1: hyphal wall protein 1
IP: immunoprecipitated
MNase: micrococcal nuclease
Ndt80: non-dityrosine 80
OD: optical density
PBS: phosphate buffered saline
PTM: post-translational modification
qPCR: quantitative PCR
Rbt1: repressed by *TUP1*
Rfx2: regulatory factor X
Rob1: regulator of biofilm 1
RT-PCR: reverse transcription PCR
SEM: scanning electron microscopy
Tec1: transposon enhancement control 1
YPD: yeast peptone dextrose
YPDU: yeast peptone dextrose supplemented with uridine
ΔCt: difference in Ct values of a gene of interest and normalization control (in this case, actin)
ΔΔCt: difference in Ct (threshold cycle) values of a gene of interest in a mutant strain compared to the wild type
