# Supplementary figures and images for: The Candida albicans biofilm gene circuit modulated at the chromatin level by a recent molecular histone innovation
Source: PLoS Biol. 2019 Aug 9;17(8):e3000422. doi: 10.1371/journal.pbio.3000422 (PMC6703697; doi:10.1371/journal.pbio.3000422)

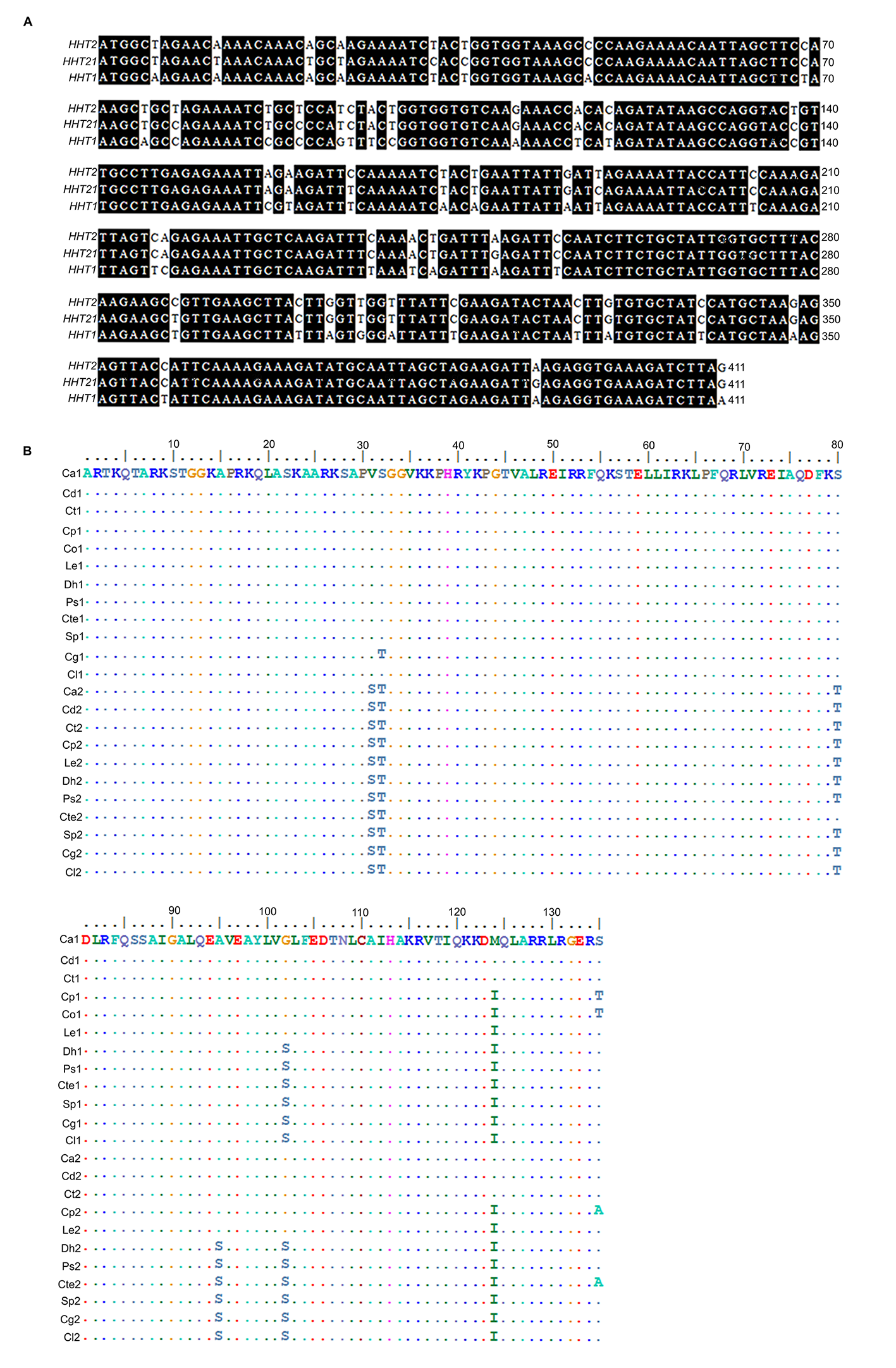

Supplement: S1 Fig — (A) Comparative analysis of nucleotide sequences of HHT2, HHT21, and HHT1, the 3 C. albicans histone H3 encoding genes. (B) Comparative analysis of the amino acid sequences of histone H3 variants of the CTG-clade species. Amino acid sequences were aligned using the Bioedit software. Identical amino acids are indicated as dots and amino acids differing from C. albicans Hht1 are given as single letter symbol: C. albicans (Ca), C. dubliniensis (Cd), C. tropicalis (Ct), C. parapsilosis (Cp), C. orthopsilosis (Co), Lodderomyces elongisporus (Le), Debaryomyces hansenii (Dh), Pichia stipitis (Ps), C. tenuis (Cte), Spathaspora passalidarum (Sp), C. guilliermondii (Cg), C. lusitaniae (Cl). Hht1 is labeled as 1 (e.g., Ca1), whereas Hht2/Hht21 is labeled as 2 (e.g., Ca2) for each species. (TIF) [file pbio.3000422.s001.tif]

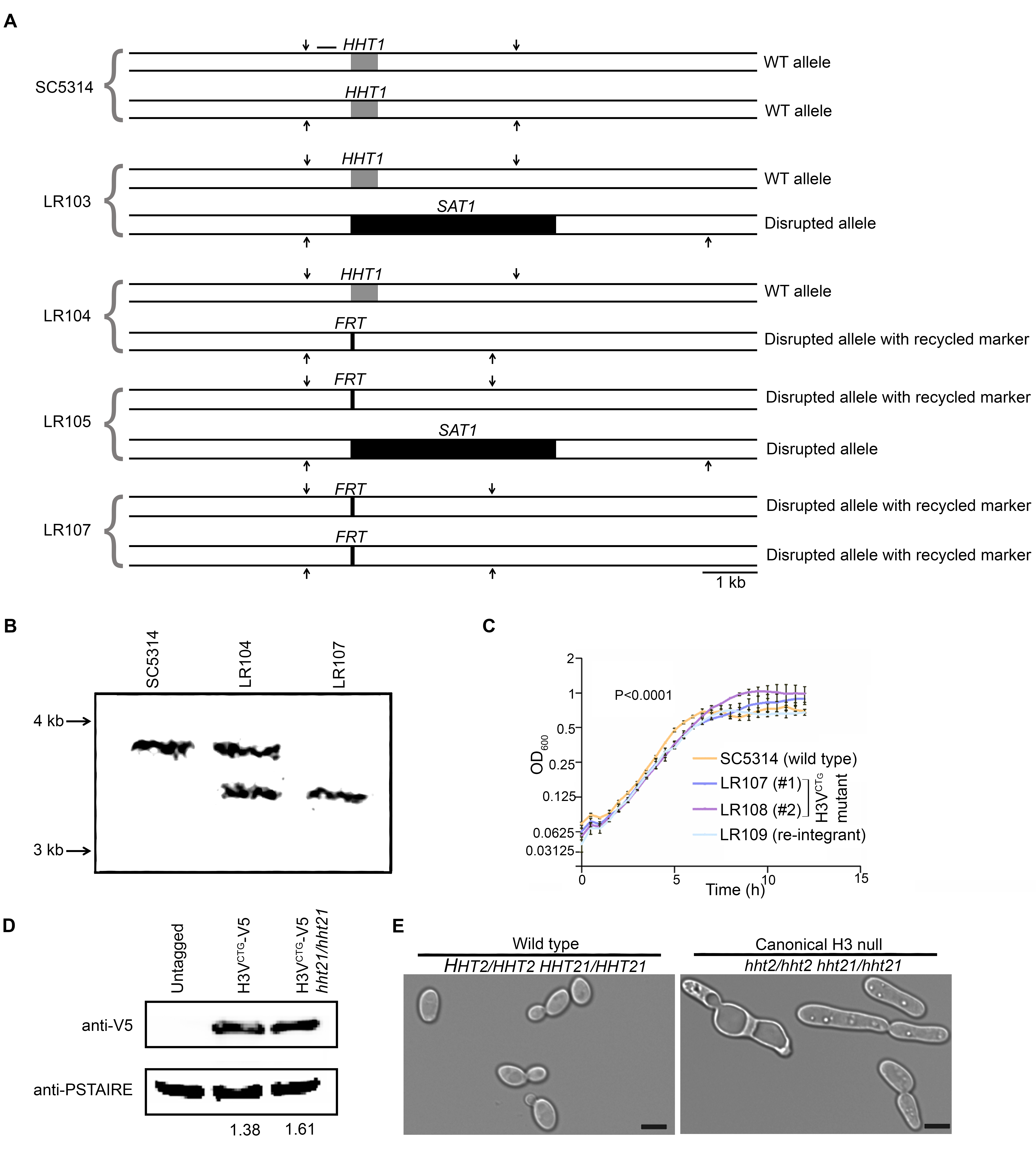

Supplement: S3 Fig — (A) Structural schematic of the HHT1 locus in the diploid C. albicans strains, namely, the wild-type SC5314 (HHT1/HHT1), heterozygous mutants (HHT1/hht1) LR103 and LR104, and homozygous null mutants LR105 and LR107 (hht1/hht1). Genomic NcoI sites are marked by down arrows. Location of the variant histone H3 gene, HHT1, is shown as a gray box, whereas the SAT1 cassette is shown as a black box. (B) Isolated genomic DNA from each indicated strain was digested with NcoI and Southern hybridized with an upstream probe (shown by the black bar in panel A). Expected results for the correct transformants were obtained. (C) Growth assays were performed by growing SC5314, LR107, LR108, and the H3VCTG complemented strain LR109 in YPDU liquid medium until the stationary phase was reached. Optical density was measured by using Varioskan Flash (Thermo Scientific). The data underlying this figure can be found in S2 Data. (D) Expression levels of histone H3 were monitored by western blot analysis of tagged strains LR144 (H3VCTG-V5) and LR149 (H3VCTG-V5 hht21/hht21) grown as yeast; the expected size of Hht1 is approximately 17 kDa. The parental strain SN148 was used as the untagged control, whereas PSTAIRE (approximately 34 kDa) was used as the loading control. Levels of histone H3 are normalized with the corresponding PSTAIRE levels, and values are indicated below each lane. (E) Wild-type (SC5314) and canonical histone H3 null mutant (LR155) strains were grown in YPD medium, and DIC images were taken at 60×. Scale bars: 2 μm. YPD, yeast peptone dextrose; YPDU, yeast peptone dextrose supplemented with uridine. (TIF) [file pbio.3000422.s003.tif]

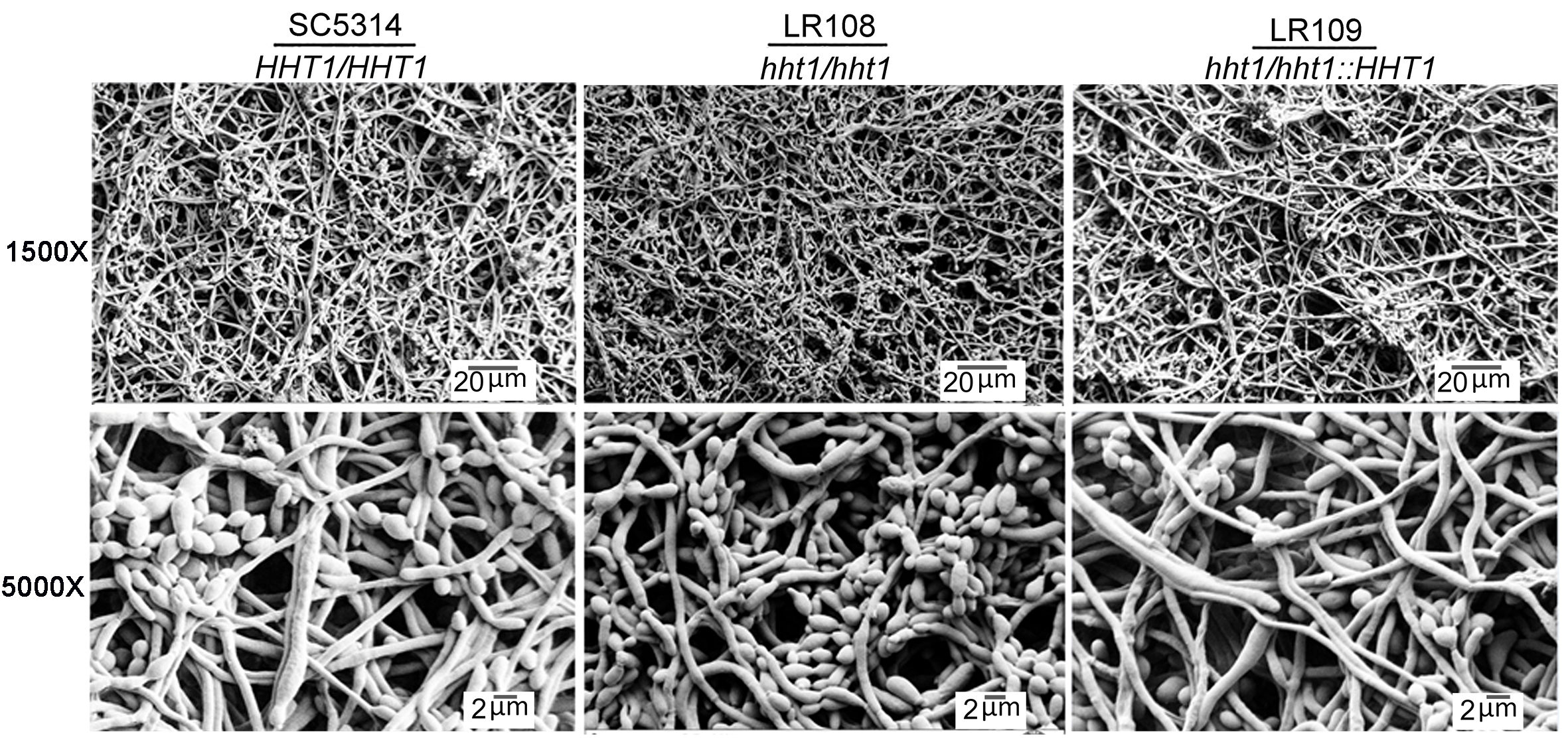

Supplement: S5 Fig — (A)Wild-type SC5314 (HHT1/HHT1), H3VCTG null mutant, LR108 (hht1/hht1), and the H3VCTG complemented strain LR109 were allowed to form biofilms on human urinary catheters for 48 hours at 37 °C. The catheter luminal surfaces were visualized by SEM. SEM, scanning electron microscopy. (TIF) [file pbio.3000422.s005.tif]

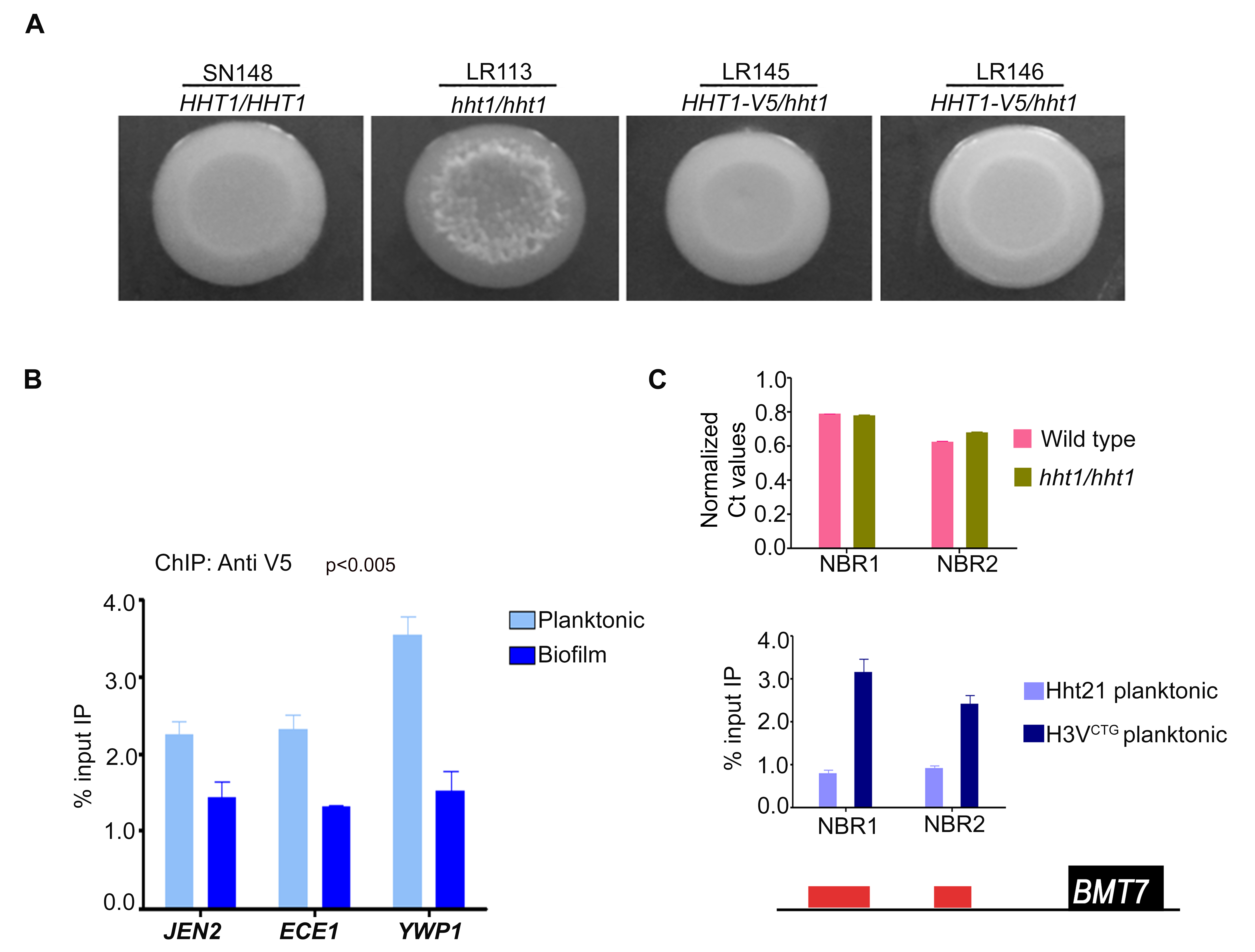

Supplement: S6 Fig — (A) To examine the functionality of V5 epitope-tagged strains, wild-type, hht1 null mutant and H3VCTG-V5/hht1 strains were spotted on Spider medium. (B) ChIP assays with anti-V5 antibodies were performed in the strain LR144 expressing H3VCTG-V5 and grown in planktonic or biofilm conditions. The enrichment of H3VCTG-V5 to the gene bodies of biofilm genes was compared in both planktonic and biofilm conditions. The data underlying this figure can be found in S2 Data. (C) MNase digestion of the genomic DNA isolated from cells of the wild-type and hht1/hht1 null mutant (LR107) grown in planktonic conditions was performed. The MNase digested DNA was precipitated and quantified by qPCR. The normalized Ct values represent the occupancy of nucleosomes at 2 previously known locations (NBR1, NBR2) at the promoter of the BMT7 gene. These 2 regions have been predicted to be nucleosome bound. Similarly, MNase ChIP was performed in LR143 (Hht21-V5) and LR144 (H3VCTG-V5) strains. A greater enrichment of H3VCTG-V5 compared to Hht21-V5 was observed at those 2 regions (NBR1, NBR2). The data underlying this figure can be found in S2 Data. ChIP, chromatin immunoprecipitation; MNase, micrococcal nuclease; qPCR, quantitative PCR. (TIF) [file pbio.3000422.s006.tif]

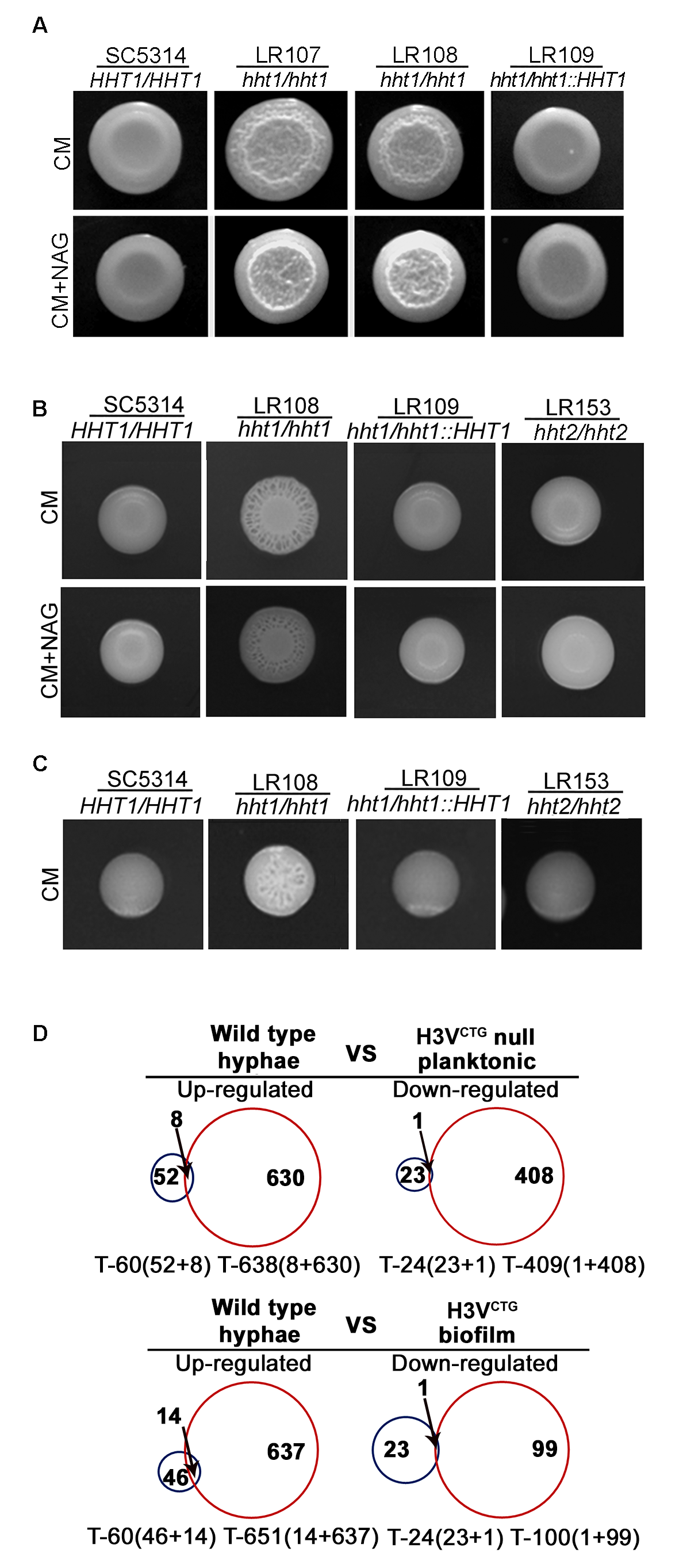

Supplement: S7 Fig — (A) SC5314, null mutants of H3VCTG (LR107, LR108), and the H3VCTG complemented strain LR109 (hht1/hht1::HHT1) were grown in liquid YPD medium and then spotted on CM and CM-containing N-acetyl glucosamine agar plates and incubated for 2 to 3 days at 30 °C. (B) SC5314, LR108, H3VCTG complemented strain LR109 (hht1/hht1::HHT1), and canonical histone H3 mutant LR153 (hht2/hht2) were grown in liquid YPD and then spotted on plates containing the indicated media and incubated for 2 to 3 days at 37 °C. (C) Similarly, the extent of filamentation was monitored for the indicated strains by growing colonies from single cells on CM medium at 37 °C. (D) Comparative analysis of filamentation-specific genes after excluding genes common between biofilm and filamentation pathways. The Venn diagrams show the comparison of differentially expressed genes between wild-type SC5314 strain grown in filamentation-induced conditions with H3VCTG null mutant grown either in planktonic or biofilm-inducing conditions. An arrow shows the overlapping genes. CM, complete media; YPD, yeast peptone dextrose. (TIF) [file pbio.3000422.s007.tif]
