# Supplementary material for: The Candida albicans biofilm gene circuit modulated at the chromatin level by a recent molecular histone innovation
Source: PLoS Biol. 2019 Aug 9;17(8):e3000422. doi: 10.1371/journal.pbio.3000422 (PMC6703697; doi:10.1371/journal.pbio.3000422)
Supplement: S1 Text — (DOCX) [file pbio.3000422.s008.docx]

**Supporting Experimental Procedures**

**Construction of *C. albicans* strains**

**Construction of LR107, LR108, LR141, LR142, LR153 and RS102:** Null mutants of variant histone H3 (*hht1/hht1*::*FRT)*, LR107 and LR108, and of canonical histone H3 encoding genes *HHT21*(LR141 and LR142) and *HHT2* (LR153 and RS102) were constructed using SC5314 as the parent strain. The *SAT1*-flipper cassette flanked by upstream and downstream sequences of *HHT1* (pLSR103), *HHT21* (pLSR106) or *HHT2* (pRS101) was digested with *Sac*I and *Kpn*I and transformed into SC5314. Transformants were selected for nourseothricin resistance. Deletion of one copy of *HHT1* was confirmed by PCR in two independent clones, LR103 and LR104. To recycle the marker, cells were grown in maltose-containing medium lacking nourseothricin, as described in [[1](#_ENREF_37)]. The loss of *SAT1* was confirmed by streaking cells on YPD and nourseothricin-containing YPD plates; two clones were selected (LR105 and LR106). To delete the remaining allele of *HHT1*, the same *SAT*-flipper cassette was used to transform LR105 and LR106. Two independent clones bearing H3V^CTG^ null mutations, LR107 and LR108, were kept. Deletion of both copies of *HHT1* was confirmed by Southern blot analysis. Similarly, deletion of one allele of *HHT21* or *HHT2* was confirmed by PCR, and the marker was recycled as before, yielding strain LR140 and LR152, respectively. The second alleles were deleted in LR140 or LR152 as described above, and two independent transformants named LR141 and LR142 (*hht21* null mutants), and LR153 and RS102 (*hht2* null mutants) were kept. Deletions were confirmed by PCR.

**Construction of a strain deleted for both canonical histone H3 encoding genes, LR155:** The canonical histone H3 gene *HHT21* was deleted in LR153 (*hht2/hht2*). A transient CRISPR-cas9 system approach was used to delete both alleles as described in [[2](#_ENREF_38)]. The *SAT1*-flipper deletion cassette from pLSR106 (see above) was co-transformed with 4µg of *CaCAS9* (obtained from pV1093 by digesting with *Kpn*1/*Apa*1 and 1µg of *HHT21*-sgRNA cassette which amplified with primer pair SgRNAFPHHT2 and SgRNARPHHT2. Deletion of both alleles of *HHT21* was confirmed by PCR.

**LR111 and LR112:** To delete both alleles of *HHT1* in *C. albicans* strain SN148, two deletion cassettes were constructed. One allele of *HHT1* was replaced by *HIS1* as follows: a 2922 bp fragment was released from the plasmid pLSR105 with *Sac*I and *Kpn*I and used to transform SN148, yielding LR110. Transformants were selected on complete media without histidine (CM-his), and the allele replacement by *HIS1* was confirmed by PCR. To delete the remaining allele of *HHT1*, the *SAT1*-flipper cassette flanked by the upstream and downstream sequences of *HHT1*of pSLR103 was released with *Sac*I and *Kpn*I and used to transform LR110 (*HHT1*/*hht1*). Transformants were selected on YPD plates containing nourseothricin. Deletion of both alleles of *HHT1* was confirmed by PCR in two independent clones named LR111 and LR112.

**LR109:** To complement the null mutant of H3V^CTG^, the *NAT1* marker of strain LR108 was recycled as above before transforming with the plasmid pLSR104 digested with *Apa*I and *Sac*I, targeting *HHT1* native locus. Transformants were selected for nourseothricin resistance, and reintegration of *HHT1* at the right locus was confirmed by PCR, yielding LR109 (*hht1/HHT1::NAT1-FLP*).

**LR113 and LR114:** The *URA3* gene was integrated at the *RPS10* locus of LR111 and LR112, using the plasmid CIp10 [[3](#_ENREF_58)], yielding strains LR113 and LR114, respectively. Correct integration of the plasmid was confirmed by PCR.

**Construction of double mutants:** The following double mutant strains: LR121 (*bcr1/bcr1 hht1/hht1*), LR123 (*brg1/brg1 hht1/hht1*), LR125 (*efg1/efg1 hht1/hht1*), LR127 (*ndt80/ndt80 hht1/hht1*), LR129 (*rob1/rob1 hht1/hht1*) and LR131 (*tec1/tec1 hht1/hht1*), were generated by deletion of both alleles of *HHT1* by the *SAT1*-flipper cassette in strains CJN688 (*bcr1/bcr1*), CJN2338 (*brg1/brg1*), CJN2302 (*efg1/efg1*), CJN2412 (*ndt80/ndt80*), CJN2408 (*rob1/rob1*) and CJN308 (*tec1/tec1*), respectively, as described above. Deletion of both alleles of *HHT1* in these strains was confirmed by PCR.

**LR133:** Strain LR133 (Bcr1-myc *hht1/hht1*) was constructed by deleting both alleles of *HHT1* in strain CJN1785 (Bcr1-Myc) using the *SAT1*-flipper cassette from pSLR103 as described above. Deletions were confirmed by PCR.

**LR143, LR144, LR145, LR146 and RS101:** For intracellular localization of the canonical or variant histone H3, encoded by *HHT21*or *HHT1*, respectively, and to study the expression of the canonical histone H3 encoded by *HHT2*, we constructed strains in which the V5 epitope coding sequence is fused to the 3’ end of the histone H3 gene. To build LR143, the Ca*HHT21*-V5 cassette was amplified using pLSR107 as a template, and two long primers: the forward primer (1061V5lFP) contains homology to the last codons of coding region of Ca*HHT21* and the reverse primer (1061SDSRP) bears homology to 3’UTR. To build LR144, the Ca*HHT1*-V5 cassette was amplified using pLSR108 as a template and two long primers: the forward primer (6791V52LFP) contains homology to Ca*HHT1* and the reverse primer (6791DSNAT1RP) homologous to 3’UTR. Strains LR145 and LR146 [*hht1*/*HHT1*-*V5::HIS1*] were constructed to check the functionality of the V5-tagged variant histone H3. To build RS101, the Ca*HHT2*-V5 cassette was amplified using pRS102 as a template and forward primer RS172 (bearing homology to Ca*HHT2*) and reverse primer RS173 (with homology to *HHT2* 3’UTR). PCR fragments were used to transform *C. albicans* strain LR113 [[4](#_ENREF_59)]. Transformants were selected for histidine prototrophy. Expression of the fusion proteins was confirmed by western blotting and indirect immuno-fluorescence microscopy using anti-V5 antibodies.

**LR149:** Both copies of *HHT21* were deleted in Hht1-V5 tagged strain LR144, as described before.

**RS103, RS104, RS105, RS106, RS107, RS108, RS109 and RS110:** The *hht1* null mutant LR108 was complemented at the locus with the mutated alleles *hht1^V31S^* (RS103 and RS104), *hht1^S32T^* (RS105 and RS106), *hht1^S80T^* (RS107 and RS108) or *hht1^V31S,S32T^* (RS109 and RS110) from plasmids pRS103, pRS105, pRS106 and pRS104, respectively. Plasmids were cut with *Apa*I and *Sac*I prior to transformation. Transformants were selected for nourseothricin resistance and reintegration of the mutated allele of *HHT1* at the right locus was confirmed by PCR.

**Plasmids construction**

**pLSR101 and pLSR102:** Either *HHT1* or *HHT21* ORF along with 5’ and 3’ untranslated region (UTR) were cloned in a TA vector. *HHT1* chromosomal region (from coordinates 57523 to 59160 of chromosome 3) was amplified with the primer pair H3.6791USFP and H3.6791DSRP, and the PCR fragment cloned into pTZ57R/T (Thermo Scientific); similarly, *HHT21* chromosomal region (from coordinates 881615 to 883328 of chromosome 1) was amplified with the primer pair 1061USFP and 1061DSRP and cloned into pTZ57R/T, yielding plasmids pLSR01 and pLSR02 respectively.

**pLSR103:** 501 bp upstream *HHT1* and 724 bp downstream *HHT1* were PCR amplified with oligos 6791USNATFP, 6791USNATRP and 6791DSNAT1FP, 6791DSNAT1RP, respectively. The upstream fragment was cloned in pSFS2A digested with *Kpn*I and *Xho*I; the downstream fragment was inserted in the resulting plasmid digested with *Sac*I and *Sac*II.

**pLSR104:** A 692 bp fragment containing the *HHT1* coding region and upstream sequences was PCR amplified with oligos 6791RES3FP and 6791RESRP3S and cloned into pSFS2A digested with *Apa*I and *Xho*I; the 724 bp PCR fragment of *HHT1* downstream region was cloned in the resulting plasmid digested with *Sac*I and *Sac*II.

**pLSR105:** A 517 bp fragment covering *HHT1* upstream region was PCR amplified with oligos H3.6791USFP and H3.6791USRP and cloned into pBluescript KSІІ (-) digested with *Sac*I and *Xba*I; a 745 bp fragment of *HHT1* downstream sequence was amplified with oligos H3.6791DSFP and H3.6791DSRP and cloned into the resulting plasmid digested with *Kpn*I and *Sal*I. Then, a 1262 bp fragment containing *HIS1* was released from pGFP-HIS1[[5](#_ENREF_60)] and cloned into the *Eco*RI site.

**pLSR106:** A 384 bp fragment of *HHT21* upstream region and a 481 bp fragment of *HHT21* downstream sequences were PCR amplified with oligos 1061USNATFP and 1061USNATRP and 1061DSNATFP and 1061DSNATRP, respectively. The upstream region was inserted in pSFS2A cut with *Kpn*I and *Xho*I, and the downstream sequences cloned in the *Sac*I/*Sac*II sites of the resulting plasmid.

**pLSR107:** A 269 bp fragment having *ORF19.1061* and downstream sequences with V5 sequence in the primers were PCR amplified and cloned into pBluescript KSII (-) having *HIS1* sequences as *SacI*/*SacII*. The cassette was confirmed by restriction digestion.

**Construction of pLSR108:** To make the plasmid pLSR108, a 451 bp having *ORF19.6791* and downstream sequences with V5 sequence in the primers were PCR amplified and cloned into pBluescipt KSII (-) having *HIS1* sequences as *SacI*/*SacII*. The cassette was confirmed by restriction digestion.

**pRS101**: A 860 bp fragment covering *HHT2* upstream region, and a 830 bp fragment containing its downstream sequences were PCR amplified using primers HHT2USNATF and HHT2USNATR, and HHT2DSNATF, HHT2DSNATR respectively. The upstream region was cloned into the *Kpn*I and *Xho*I of pSFS2A, the downstream region was inserted into the *Sac*I/*Sac*II sites of the resulting plasmid.

**pRS102:** To make the plasmid pRS102, a 333 bp having *ORF19.1853* and downstream sequences with V5 sequence in the primers were PCR amplified and cloned into pBluescript KSII (-) having *HIS1* sequences as *SacI*/*SacII*. The cassette was confirmed by restriction digestion.

**pRS103, pRS104, pRS105 and pRS106**: The *HHT1* coding region was mutated by overlap PCR using primers sdm1fp, sdm1rp (V31S, in pRS103), primers RS181 and RS182 (V31S, S32T, in pRS104), primers RS211 and 212 (S32T, pRS105), or primers RS233 and 234 (S80T, in pRS106). A1656 bp fragment containing the mutated ORFs was cloned into the *Apa*I/*Xho*I sites of pRS107. Clones were confirmed by restriction analysis and the presence of the mutation was confirmed by Sanger sequencing.

**Biofilm measurement by Standard Optical Density Assays**: We performed 24-well Standard Optical Density Assays for single and double mutants of biofilm regulators as well as variant histone H3 *C. albicans* strains. First, strains were grown at 30°Cin YPD medium. 1.5 ml of a cell at final OD_600_=0.5 in YPD were added to the wells. The cells were allowed to form biofilms at 37°C for 24 h. Optical Density was measured using Tecan I control infinite 200 as described in [[6](#_ENREF_61)]. The average density of reads at nine independent locations in each well of the 24-well plate was calculated.

***In vitro* scanning electron microscopy (SEM) of biofilm**

SEM analysis was done with a minor modification in the protocol described earlier [[7](#_ENREF_62)]. Wild type and mutant biofilms were grown in YNB medium containing 500 mM of galactose on a human urinary catheter for 48 h and then processed for SEM imaging. Biofilms were treated with in 4% [vol/vol] formaldehyde followed by a wash with 1% [vol/vol] phosphate buffer. This was followed by the treatment with 1% osmium tetroxide (Sigma) for 1 h. Samples were subsequently washed in distilled water once and dehydrated in a series of ethanol washes (70% for 10 min, 95% for 10 min, and 100% for 20 min). Biofilms were air-dried in a desiccator prior to sputter coating with gold nanoparticles. Afterwards, the surface topographies of biofilms were visualized by taking SEM images in the high-vacuum mode.

**References**

1. Reuss O, Vik A, Kolter R, Morschhauser J. The SAT1 flipper, an optimized tool for gene disruption in Candida albicans. Gene. 2004;341:119-27. Epub 2004/10/12. doi: S0378111904003555 [pii]

10.1016/j.gene.2004.06.021. PubMed PMID: 15474295.

2. Min K, Ichikawa Y, Woolford CA, Mitchell AP. Candida albicans Gene Deletion with a Transient CRISPR-Cas9 System. mSphere. 2016;1(3). Epub 2016/06/25. doi: 10.1128/mSphere.00130-16

3. Murad AM, Lee PR, Broadbent ID, Barelle CJ, Brown AJ. CIp10, an efficient and convenient integrating vector for *Candida albicans*. Yeast. 2000;16(4):325-7. Epub 2000/02/12. doi: 10.1002/(SICI)1097-0061(20000315)16:4<325::AID-YEA538>3.0.CO;2-# [pii]

10.1002/1097-0061(20000315)16:4<325::AID-YEA538>3.0.CO;2-#. PubMed PMID: 10669870.

4. Noble SM, Johnson AD. Strains and strategies for large-scale gene deletion studies of the diploid human fungal pathogen *Candida albicans*. Eukaryot Cell. 2005;4(2):298-309. Epub 2005/02/11. doi: 4/2/298 [pii]

10.1128/EC.4.2.298-309.2005. PubMed PMID: 15701792; PubMed Central PMCID: PMC549318.

5. Gerami-Nejad M, Berman J, Gale CA. Cassettes for PCR-mediated construction of green, yellow, and cyan fluorescent protein fusions in *Candida albicans*. Yeast. 2001;18(9):859-64. Epub 2001/06/28. doi: 10.1002/yea.738. PubMed PMID: 11427968.

6. Lohse MB, Gulati M, Valle Arevalo A, Fishburn A, Johnson AD, Nobile CJ. Assessment and Optimizations of *Candida albicans* *In Vitro* Biofilm Assays. Antimicrob Agents Chemother. 2017;61(5). Epub 2017/03/16. doi: AAC.02749-16 [pii]

10.1128/AAC.02749-16. PubMed PMID: 28289028.

7. Samaranayake YH, Ye J, Yau JY, Cheung BP, Samaranayake LP. In vitro method to study antifungal perfusion in Candida biofilms. J Clin Microbiol. 2005;43(2):818-25. Epub 2005/02/08. doi: 43/2/818 [pii]

10.1128/JCM.43.2.818-825.2005. PubMed PMID: 15695686; PubMed Central PMCID: PMC548120.
