# Supplementary material for: The Candida albicans biofilm gene circuit modulated at the chromatin level by a recent molecular histone innovation
Source: PLoS Biol. 2019 Aug 9;17(8):e3000422. doi: 10.1371/journal.pbio.3000422 (PMC6703697; doi:10.1371/journal.pbio.3000422)
Supplement: S1 Table — (DOCX) [file pbio.3000422.s009.docx]

**S1 Table. Histone H3 gene polymorphism in *C. albicans***

| Gene | Nucleotide  position in ORF | Amino acid  position in protein | Amino acid change | Hap A allele | Genotypes (frequencies in %) |
| --- | --- | --- | --- | --- | --- |
| *HHT1* | 270 | 90 | Synonymous | T | TT (86.8), TC (11.0), CC (2.2) |
| *HHT1* | 303 | 101 | Synonymous | A | AA (99.5), AG (0.5) |
| *HHT2* | 138 | 46 | Synonymous | T | TT (82.9), TC (13.8), CC (3.3) |
| *HHT2* | 189 | 63 | Synonymous | T | TT (97.3), TC (2.7) |
| *HHT2* | 195 | 65 | Synonymous | A | AA (91.2), AG (8.8) |
| *HHT2* | 298 | 100 | Y 🡪 N | T | TT (99.5), TA (0.5) |
| *HHT2* | 393 | 131 | Synonymous | G | AA (48.9), GA (43.9), GG (7.2) |
| *HHT21* | 36 | 12 | Synonymous | C | CC (99.5), CT (0.5) |
| *HHT21* | 63 | 21 | Synonymous | A | AA (97.8), AG (1.1), GG (1.1) |
| *HHT21* | 87 | 29 | Synonymous | T | TT (99.5), TG (0.5) |
| *HHT21* | 165 | 55 | Synonymous | T | CC (33.3), TC (33.9), TT (32.8) |
| *HHT21* | 177 | 59 | Synonymous | T | TT (97.8), TC (1.1), CC (1.1) |
| *HHT21* | 288 | 96 | Synonymous | T | TT (50.9), TC (36.4), CC (10.4), CG (1.7), GG (0.6) |
| *HHT21* | 296 | 99 | A 🡪 V | C | CC (98.9), CT (1.1) |
| *HHT21* | 330 | 110 | Synonymous | G | GG (86.1), GA (11.7), AA(2.2) |
| *HHT21* | 357 | 119 | Synonymous | C | CC (96.7), CA (3.3) |
| *HHT21* | 406 | 136 | S 🡪 A | T | TT (99.5), GG (0.5) |
