# Supplementary material for: The Candida albicans biofilm gene circuit modulated at the chromatin level by a recent molecular histone innovation
Source: PLoS Biol. 2019 Aug 9;17(8):e3000422. doi: 10.1371/journal.pbio.3000422 (PMC6703697; doi:10.1371/journal.pbio.3000422)
Supplement: S2 Table — (DOCX) [file pbio.3000422.s010.docx]

**S2 Table. *C. albicans* strains and plasmids used in this study**

| **Yeast strain** | **Genotype** | **Reference** |
| --- | --- | --- |
| SC5314 | Wild-type clinical isolate | [1] |
| SN148 | ∆ura3::imm434/∆ura3::imm434 ∆his1::hisG/∆his1::hisG, ∆arg4::hisG/∆arg4::hisG, ∆leu2:: hisG/∆leu2::hisG | [2] |
| LR103 | SC5314 hht1NATflp/HHT1 | This study |
| LR104 | SC5314 ∆hht1NATflp/HHT1 | This study |
| LR105 | SC5314 HHT1/∆hht1::*FRT* | This study |
| LR106 | SC5314 HHT1/∆hht1::*FRT* | This study |
| LR107 | SC5314 ∆hht1::FRT/∆hht1::*FRT* | This study |
| LR108 | SC5314 ∆hht1::FRT/∆hht1::*FRT* | This study |
| LR109 | SC5314 ∆hht1::FRT/∆hht1::HHT1::FRT | This study |
| LR110 | SN148 ∆hht1::HIS1/HHT1 | This study |
| LR111 | SN148 ∆hht1::HIS1/∆hht1::FRT | This study |
| LR112 | SN148 ∆hht1::HIS1/∆hht1::FRT | This study |
| LR113 | SN148 ∆hht1::HIS1/∆hht1::FRT RPS10/rps10::URA3 | This study |
| LR114 | SN148 ∆hht1::HIS1/∆hht1::FRT RPS10/rps10::URA3 | This study |
| CJN308 | ∆ura3::imm434/∆ura3::imm434 ∆his1::hisG/∆his1::hisG, ∆arg4::hisG/∆arg4::hisG tec1::Tn7-UAU1/tec1::Tn7URA3 | [3] |
| CJN688 | ∆ura3::imm434/∆ura3::imm434 ∆his1::hisG/∆his1::hisG, ∆arg4::hisG/∆arg4::hisG ∆bcr1::ARG4/∆bcr1::URA3 | [3] |
| CJN2302 | ∆ura3::imm434/∆ura3::imm434/URA3-IRO1 ∆his1::hisG/∆his1::hisG ∆arg4::hisG/∆arg4::hisG ∆leu2::hisG/∆leu2::hisG::cdARG4 ∆efg1::CmLEU2/∆efg1::CdHIS1 | [4] |
| CJN2338 | ∆ura3::imm434/∆ura3::imm434/URA3-IRO1 ∆his1::hisG/∆his1::hisG ∆arg4::hisG/∆arg4::hisG ∆leu2::hisG/∆leu2::hisG::CdARG4 ∆brg1::CmLEU2/∆brg1::CdHIS1 | [4] |
| CJN2408 | ∆ura3::imm434/∆ura3::imm434/URA3-IRO1 ∆his1::hisG/∆his1::hisG ∆arg4::hisG/∆arg4::hisG ∆leu2::hisG/∆leu2::hisG::CdARG4 ∆rob1::CmLEU2/∆rob1::CdHIS1 | [4] |
| CJN2412 | ∆ura3::imm434/∆ura3::imm434/URA3-IRO1 ∆his1::hisG/∆his1::hisG ∆arg4::hisG/∆arg4::hisG ∆leu2::hisG/∆leu2::hisG::cdARG4 ∆ndt80::CmLEU2/∆ndt80::CdHIS1 | [4] |
| LR121 | ∆ura3::imm434/∆ura3::imm434 ∆his1::hisG/∆his1::hisG ∆arg4::hisG/∆arg4::hisG ∆bcr1::ARG4/∆bcr1::URA3 ∆hht1::FRT/∆hht1::FRT | This study |
| LR123 | ∆ura3::imm434/∆ura3::imm434/URA3-IRO1 ∆his1::hisG/∆his1::hisG ∆arg4::hisG/∆arg4::hisG ∆leu2::hisG/∆leu2::hisG::CdARG4 ∆brg1::CmLEU2/∆brg1::CdHIS1 ∆hht1::FRT/∆hht1::FRT | This study |
| LR125 | ∆ura3::imm434/∆ura3::imm434/URA3-IRO1 ∆his1::hisG/∆his1::hisG ∆arg4::hisG/∆arg4::hisG ∆leu2::hisG/∆leu2::hisG::CdARG4 ∆efg1::CmLEU2/∆efg1::CdHIS1 ∆hht1::FRT/∆hht1::FRT | This study |
| LR127 | ∆ura3::imm434/∆ura3::imm434/URA3-IRO1 ∆his1::hisG/∆his1::hisG, ∆arg4::hisG/∆arg4::hisG, ∆leu2::hisG/∆leu2::hisG::CdARG4 ∆ndt80::CmLEU2/∆ndt80::CdHIS1 ∆hht1::FRT/∆hht1 ::FRT | This study |
| LR129 | ∆ura3::imm434/∆ura3::imm434/URA3-IRO1 ∆his1::hisG/∆his1::hisG ∆arg4::hisG/∆arg4::hisG ∆leu2::hisG/∆leu2::hisG::CdARG4 ∆rob1::CmLEU2/∆rob1::CdHIS1 ∆hht1::FRT/∆hht1::FRT | This study |
| LR131 | ∆ura3::imm434/∆ura3::imm434 ∆his1::hisG/∆his1::hisG ∆arg4::hisG/∆arg4::hisG tec1::Tn7-UAU1/tec1::Tn7URA3 ∆hht1::FRT/∆hht1::FRT | This study |
| CJN1785 | ∆ura3:: imm434/∆ura3::imm434/URA3-IRO1 ∆his1::hisG/∆his1::hisG ∆arg4::hisG/∆arg4::hisG ∆leu2::hisG::pHIS1/∆leu2::hisG::BCR1-13XMyc-FRT | [4] |
| LR132 | ∆ura3:: imm434/∆ura3::imm434/URA3-IRO1 ∆his1::hisG/∆his1::hisG ∆arg4::hisG/∆arg4::hisG ∆leu2::hisG::pHIS1/∆leu2::hisG::BCR1-13XMyc-FRT HHT1 /hht1::NAT FLP | This study |
| LR133 | ∆ura3:: imm434/∆ura3::imm434/URA3-IRO1 ∆his1::hisG/∆his1::hisG ∆arg4::hisG/∆arg4::hisG ∆leu2::hisG::pHIS1/∆leu2::hisG::BCR1-13XMyc-FRT hht1::FRT/hht1::NAT FLP | This study |
| LR141 | ∆hht21::FRT /∆hht21::*FRT* | This study |
| LR142 | ∆hht21::FRT /∆hht21::*FRT* | This study |
| LR143 | ∆ura3:: imm434/∆ura3::imm434 ∆his1::hisG/∆his1::hisG ∆arg4::hisG/∆arg4::hisG ∆leu2:: hisG/∆leu2::hisG HHT21/HHT21::V5-HIS1 RPS10/rps10::URA3 | This study |
| LR144 | ∆ura3:: imm434/∆ura3::imm434 ∆his1::hisG/∆his1::hisG ∆arg4::hisG/∆arg4::hisG ∆leu2:: hisG/∆leu2::hisG HHT1/HHT1::V5-HIS1 RPS10/rps10::URA3 | This study |
| LR145 | ∆ura3:: imm434/∆ura3::imm434 ∆his1::hisG/∆his1::hisG ∆arg4::hisG/∆arg4::hisG ∆leu2::hisG/∆leu2::hisG ∆hht1::NAT FLP/HHT1::V5 -HIS1 RPS10/rps10::URA3 | This study |
| LR146 | ∆ura3:: imm434/∆ura3::imm434 ∆his1::hisG/∆his1::hisG ∆arg4::hisG/∆arg4::hisG ∆leu2::hisG/∆leu2::hisG ∆hht1::NAT FLP/HHT1::V5 -HIS1 RPS10/rps10::URA3 | This study |
| LR148 | ∆ura3:: imm434/∆ura3::imm434 ∆his1::hisG/∆his1::hisG ∆arg4::hisG/∆arg4::hisG ∆leu2:: hisG/∆leu2::hisG HHT1/HHT1::V5-HIS1 HHT21/∆hht21::*FRT* RPS10/rps10::URA3 | This study |
| LR149 | ∆ura3:: imm434/∆ura3::imm434 ∆his1::hisG/∆his1::hisG ∆arg4::hisG/∆arg4::hisG ∆leu2:: hisG/∆leu2::hisG HHT1/HHT1::V5 HIS1 ∆hht21::FRT/∆hht21::*FRT* RPS10/rps10::URA3 | This study |
| RS101 | ∆ura3::imm434/∆ura3::imm434 ∆his1::hisG/∆his1::hisG ∆arg4::hisG/∆arg4::hisG ∆leu2:: hisG/∆leu2::hisG HHT2/HHT2::V5 HIS1 RPS10/rps10::URA3 | This study |
| LR152 | SC5314 HHT2/∆hht2::*FRT* | This study |
| LR153 | SC5314 ∆hht2::FRT/∆hht2::*FRT* | This study |
| RS102 | SC5314 ∆hht2::FRT/∆hht2::*FRT* | This study |
| RS103 | ∆hht1::FRT/∆hht1::HHT1^V31S^ NAT-FLP | This study |
| RS104 | ∆hht1::FRT/∆hht1::HHT1^V31S^ NAT-FLP | This study |
| RS105 | ∆hht1::FRT/∆hht1::HHT1^S32T^ NAT-FLP | This study |
| RS106 | ∆hht1::FRT/∆hht1::HHT1^S32T^ NAT-FLP | This study |
| RS107 | ∆hht1::FRT/∆hht1::HHT1^S80T^ NAT-FLP | This study |
| RS108 | ∆hht1::FRT/∆hht1::HHT1^S80T^ NAT-FLP | This study |
| RS109 | ∆hht1::FRT/∆hht1::HHT1^VS3132ST^ NAT-FLP | This study |
| RS110 | ∆hht1::FRT/∆hht1::HHT1^VS3132ST^ NAT-FLP | This study |
| LR155 | ∆hht21::FRT/∆hht21::*FRT* ∆hht2::SAT1/∆hht2::*SAT1* | This study |
| **Plasmid** | **Description** | **References** |
| pLSR01 | pTZ57R/T + CaHHT1 | This study |
| pLSR02 | pTZ57R/T + CaHHT21 | This study |
| pLSR103 | pSFS2A + 5’CaHHT1 + 3’CaHHT1 | This study |
| pLSR104 | pSFS2A + 5’CaHHT1 + CaHHT1+3’CaHHT1 | This study |
| pLSR105 | pBluescript + 5’CaHHT1 +CaHIS1 +3’CaHHT1 | This study |
| pLSR106 | pSFS2A + 5’CaHHT21 + 3’CaHHT21 | This study |
| pLSR107 | pBluescript + CaHHT21+V5 +CaHIS1 +3’CaHHT21 | This study |
| pLSR108 | pBluescript + CaHHT1+V5 +CaHIS1 +3’CaHHT1 | This study |
| CIp10 | pBluescript + CaURA3 +RP10 | [5] |
| pRS101 | pSFS2A + 5’CaHHT2 + 3’CaHHT2 | This study |
| pRS102 | pBluescript + CaHHT2+V5 +CaHIS1 +3’CaHHT2 | This study |
| pRS103 | pSFS2A + 5’CaHHT1 + CaHHT1V31S+3’CaHHT1 | This study |
| pRS104 | pSFS2A + 5’CaHHT1 +CaHHT1VS3132ST+3’CaHHT1 | This study |
| pRS105 | pSFS2A + 5’CaHHT1 + CaHHT1S32T+3’CaHHT1 | This study |
| pRS106 | pSFS2A + 5’CaHHT1 + CaHHT1S80T+3’CaHHT1 | This study |
| pRS107 | pSFS2A + 3’CaHHT1 | This study |

**References**

1. Gillum AM, Tsay EY, Kirsch DR (1984) Isolation of the *Candida albicans* gene for orotidine-5'-phosphate decarboxylase by complementation of *S. cerevisiae ura3* and *E. coli* pyrF mutations. Mol Gen Genet 198: 179-182.

2. Noble SM, Johnson AD (2005) Strains and strategies for large-scale gene deletion studies of the diploid human fungal pathogen *Candida albicans*. Eukaryot Cell 4: 298-309.

3. Nobile CJ, Mitchell AP (2005) Regulation of cell-surface genes and biofilm formation by the *C. albicans* transcription factor Bcr1p. Curr Biol 15: 1150-1155.

4. Nobile CJ, Fox EP, Nett JE, Sorrells TR, Mitrovich QM, et al. (2012) A recently evolved transcriptional network controls biofilm development in *Candida albicans*. Cell 148: 126-138.

5. Murad AM, Lee PR, Broadbent ID, Barelle CJ, Brown AJ (2000) CIp10, an efficient and convenient integrating vector for *Candida albicans*. Yeast 16: 325-327.
