# Supplementary material for: The Candida albicans biofilm gene circuit modulated at the chromatin level by a recent molecular histone innovation
Source: PLoS Biol. 2019 Aug 9;17(8):e3000422. doi: 10.1371/journal.pbio.3000422 (PMC6703697; doi:10.1371/journal.pbio.3000422)
Supplement: S3 Table — (DOCX) [file pbio.3000422.s011.docx]

**S3 Table. Oligonucleotide primers used in this study**

| **Primer name** | **Sequence (5’-3’)** |
| --- | --- |
| H3.2 | TGCCAGAAAATCTGCCCCATCTACT |
| H3-4 | ACAGCTTCTTGTAAAGCACCAATAGCAG |
| H3-7 | AGCCAGAAAATCCGCCCCAGTTTCC |
| H3.6791USFP | CGAGCTCTTTTTTTGCTAGAGTATGCA |
| H3.6791USRP | GCTCTAGATGCCATTATTGATTAAAAGT |
| H3.6791DSFR | ACGCGTCGACAAGACAGGATAAGATAGGATAGG |
| H3.6791DSRP | GGGGTACCGTTGGTTTGTTTGTTAGTTAG |
| 1061USFP | CGAGCTCGACTAAAATGAAAGTAAAACAATAAAGC |
| 1061DSRP | GGGGTACCCGGTTGGTATACCGCTGATGAATCT |
| 1061V52CFP | CGA GCT CTT GAG AGG TGA AAG ATC TGG TAA GCC TAT CCC TAA CCC TCT CCT CGG TCT CGA TTC TAC GTA GGT TAA GCT CGT GGC GGG |
| 1061V5CRP | TCC CCG CGG CCG GAT GTT TGT TTT ATT TTT TTC |
| 1061V5lFP | TAA CTT GTG TGC TAT CCA TGC TAA GAG AGT TAC CAT TCA AAA GAA AGA TAT GCA ATT AGC TAG AAG ATT GAG AGG TGA AAG ATC T |
| 6791V52CFP | CGA GCT CTT AAG AGG TGA AAG ATC TGG TAA GCC TAT CCC TAA CCC TCT CCT CGG TCT CGA TTC TAC GTA AGA CAG GAT AAG ATA GGA T |
| 6791V5CRP | TCC CCG CGG GAC TTC AAG ATT ATA ATT AAA ACA AAG |
| 6791V52LFP | CTA ATT TAT GTG CTA TTC ATG CTA AAA GAG TTA CTA TTC AAA AGA AAG ATA TGC AAT TAG CTA GAA GAT TAA GAG GTG AAA GAT CT |
| H3.US | CGGAGTTAGATAAGCCGCCCG |
| H3.HIS | CCTGCTTAACTTCTTTCGACCACG |
| 1061SDSFP | GTTTTATGGATTATTGTTTTTAA |
| 1061SDSRP | GAAGAACCTTATATCCCAAGAAG |
| 6791SUSFP | CTCCAGCTCCATGGGCAAATAC |
| 6791SUSFP | TAGGTCAGATCAGGTCAGGTC |
| HHT1FP | CTT CTC CTT ATA CTT ATT TAA C |
| HHT1RP | CCA CCG GAA ACT GGG GCG |
| HHT21FP | TTT TTA TAT ATC ATC TAA ACA TTC |
| HHT21RP | GAC ACC ACC AGT AGA TGG G |
| 1061USNATFP | GGGGTACCTCCATTCCTTCCTGTCT |
| 1061USNATRP | CCGCTCGAGTGTTGTGTGTGATAGAATG |
| 1061DSNATFP | TCCCCGCGGGTTAAGCTCGTGGCGGG |
| 1061DSNATRP | CGAGCTCGTTGGTGAATACAAGGGT |
| 6791USNATFP | GGG GTA CCC TAG AGT ATG CAC TAC TTT TT |
| 6791USNAT1RP | CCG CTC GAG TAT TGA TTA AAA GTG TTG TTA |
| 6791DSNAT1FP | TTC CCG CGG GAC AGG ATA AGA TAG GAT AG |
| 6791DSNAT1RP | CGA GCT CGA TAA GGT TGG TTT GTT TGT TAG |
| 6791RES3FP | TAA GGG CCC CTA TTT CAT TTG CTC TCT CC |
| 6791RESRP3S | CCG CTC GAG TTA AGA TCT TTC ACC TC |
| HWP1RTFP | CCG CTC GAG TTA AGA TCT TTC ACC TC |
| HWP1RTRP | GAA ATA GGA GCG ACA CTT G |
| ALS3RTFP | CGC AAT CCA ATT CTG ATA CC |
| ALS3RTRP | GAA TAA CAG AAC CAG ATC CG |
| ECE1FP | CAA CCA GTT AAA AGA GAT GCC |
| ECE1RP | TTT CTG AAA CAA TTT GAG CAG C |
| YWP1FP | GTTGCTGGTGGTGTTAATGG |
| YWP1RP | AAG TAC TAA TGG CAG CTT TAC C |
| GCA1FP | GGT GAA TAT GAA GTT CGT CAA CC |
| GCA1RP | GTA GTT GTG GCT TAC TGT TTC G |
| JEN2FP | TTT ATT GGT CCT GAA AAC AGA GG |
| JEN2RP | GAA TCA CCT CTG TCT TCC CTA TC |
| ALD6FP | GGT AAG GCT GGT ATT ACT TTC TTG |
| ALD6RP | TTG GCA TTG AAG TAG AAG GTT TC |
| MET3RTFP | CAA TCA AGC TTC TGG TAA TGC |
| MET3RTRP | CAC AAT TTC ATC AAC TAC AGC |
| HWP1PFP2 | CCC TTA AAA CCG ATC AAG AAA G |
| HWP1PRP2 | CGA GAC GAG GAC AAC AAC |
| ECE1PFP2 | GGT GAT GAA TGG TGA TTG AAT G |
| ECE1PRP2 | GTG TCA ATT TTA CGG CTT TGT |
| YWP1P2FP | TTG ATA CTA TTT CCT CAA AAA GCC |
| YWP1P2RP | GTT TCT AAA AGA GGC GTT GCT G |
| NRG1PFP | CAT ATT GGT GTA TAA TAA TCA TC |
| NRG1PRP | AAA CAA CAC CAT ACA ATG TGA CAC |
| CAN1PFP | TCC GAA TTG ATA TCT CGT TTA AG |
| CAN1PRP | TGG AAG AGA TGA GCC AGT GGT G |
| BMT7PFP | TTG AAC TAA AAG GCT GAG CAT G |
| BMT7PRP | ATA TAT GCG ATG GAT TAG TCA TC |
| HGT2PFP | TTT GGT CTA GCT GGG GGG |
| HGT2PRP | CAA AGC ACA CAT TAA TAT CCA GC |
| SAP5PFP | ACG CAA TTT CAC CAA TTA TAG TC |
| SAP5PRP | GGC AGG TTT GTA AGT AAA TAA TG |
| JEN2PFP | GAG TTT TGT GTA ATG ACC AGC |
| JEN2PRP | TCA CTT TGT TGT ATT TTG TGG |
| Orf19.7380PFP | CCT CAA TTA CCT TGC AGT AGT C |
| Orf19.7380PRP | CAA TCA AGT ACA GCG CAA AC |
| 1853FP | CGG TAC CTG ACA TTG TTG ATT GA |
| HHT2USNATF | CGGGGTACCGAAAGAATTATAATCCAA |
| HHT2USNATR | CGGGGTACCGAAAGAATTATAATCCAA |
| HHT2DSNATF | TCCCCGCGGGTTTTATTTCCTTGATGG |
| HHT2DSNATR | CGAGCTCATTTGTTGCCATTGAAATGGG |
| RS170:1853v5cfp | CGAGCTCGATTGAGAGGTGAAAGATCTGGTAAGCCTATCCCTAACCCTCTCCTCGGTCTCGATTCTACGTAGGTTTTATTTCCTTGATGG |
| RS171:1853v5crp | TCCCCGCGGCATAAATAGAATTAGTCTG |
| RS172:1853ampfp | GCTATCCATGCTAAGAGAGTTACCATTCAAAAGAAAGATATGCAATTAGCTAGAAGATTGAGAGGTGAAAGATCT |
| RS173:1853amprp | CCTTTGAGAACAAGAGATCCCCCTAATGCAGCAGCAGGAATAGGTGATACCGATAATTTATCAATTGAATGGGGATCCTGGAGGATGAGGAG |
| RS180 | CCGGGGCCCGTTTTCATCCCCCAAAAAATC |
| RS185 | CCGCTCGAGGATTATCAACTCGGGGGAC |
| RS181 | GCCAGAAAATCCGCCCCATCTACTGGTGGTGTCAAAAAACC |
| RS182 | GGTTTTTTGACACCACCAGTAGATGGGGCGGATTTTCTGGC |
| Sdm1fp | GCCAGAAAATCCGCCCCATCATCCGGTGGTGTCAAAAAA |
| Sdm1rp | TTTTTTGACACCACCGGATGATGGGGCGGATTTTCTGGC |
| RS186 | TCCCCGCGGGACGAAGAATAATCTACTC |
| RS187 | CGAGCTCCATTCACACATTAATGGC |
| RS211 | CAGAAAATCCGCCCCAGTTACTGGTGGTGTCAAAAAACCTC |
| RS212 | CAGAAAATCCGCCCCAGTTACTGGTGGTGTCAAAAAACCTC |
| RS233 | GAGAAATTGCTCAAGATTTTAAAACTGATTTAAGATTTCAATCTTCTGC |
| RS234 | GCAGAAGATTGAAATCTTAAATCAGTTTTAAAATCTTGAGCAATTTCTC |
| SgRNAFPHHT2 | ACCAACCAAGTAAGCTTCAAGTTTTAGAGCTAGAAATAGCAAGTTAAA |
| SgRNARPHHT2 | TTGAAGCTTACTTGGTTGGTCAAATTAAAAATAGTTTACGCAAGTC |
